# Evaluation of antimicrobial susceptibility testing methods for *Burkholderia cepacia* complex isolates from people with and without cystic fibrosis

**DOI:** 10.1128/jcm.01480-24

**Published:** 2025-01-22

**Authors:** Peter Jorth, Carmila Manuel, Tracey McLemore, Romney M. Humphries, Nicolynn C. Cole, Audrey N. Schuetz, Dennis Garica, Maria Maldonado, Natasha Rivero, Anna Clara Milesi Galdino, Diana Celedonio, John J. LiPuma, Daniel A. Green, James E. A. Zlosnik, Maria Traczewski, Holly K. Huse

**Affiliations:** 1 Department of Pathology and Laboratory Medicine, Cedars-Sinai Medical Center 548288 , Los Angeles, California, USA; 2 Department of Medicine, Cedars-Sinai Medical Center 542818 , Los Angeles, California, USA; 3 Department of Biomedical Sciences, Cedars-Sinai Medical Center 22494 , Los Angeles, California, USA; 4 Department of Pathology, Microbiology, & Immunology, Vanderbilt University Medical Center 204907 , Nashville, Tennessee, USA; 5 Department of Laboratory Medicine and Pathology, Mayo Clinic 195112 , Rochester, Minnesota, USA; 6 Department of Pathology, Harbor-UCLA Medical Center 21640 , Torrance, California, USA; 7 Department of Pediatrics, University of Michigan 199683 , Ann Arbor, Michigan, USA; 8 Department of Pathology, New York-Presbyterian/Columbia University Medical Center 21611 , New York, New York, USA; 9 Department of Pediatrics, The University of British Columbia 195987 , Vancouver, British Columbia, Canada; 10 Clinical Microbiology Institute, Inc., Wilsonville, Oregon, USA; Children's Hospital Los Angeles, Los Angeles, California, USA

**Keywords:** *Burkholderia cepacia* complex, susceptibility testing, cystic fibrosis, antimicrobial agents

## Abstract

The *Burkholderia cepacia* complex (BCC) is a group of Gram-negative bacteria that cause opportunistic infections, most notably in people with cystic fibrosis (CF), and have been associated with outbreaks caused by contaminated medical products. Antimicrobial susceptibility testing (AST) is often used to guide treatment for BCC infections, perhaps most importantly in people with CF who are being considered for lung transplant. However, recent studies have highlighted problems with AST methods. Here, we address limitations from previous studies to further evaluate BCC AST methods. We assessed the performance of reference broth microdilution (BMD), disk diffusion (DD) using Mueller-Hinton agar (MHA) from three manufacturers, agar dilution (AD), and gradient diffusion (ETEST) for ceftazidime (CAZ), levofloxacin (LVX), meropenem (MEM), minocycline (MIN), and trimethoprim-sulfamethoxazole (TMP-SMX) on a set of 205 BCC isolates. The isolate set included 100 isolates from people with CF and 105 isolates from people without CF from a variety of sources, which enabled us to systematically evaluate whether specimen source impacts AST performance. For all BCC isolates, BMD reproducibility was 93%, 98%, 99%, 98%, and 96% for CAZ, LVX, MEM, MIN, and TMP-SMX, respectively. Using BMD as the comparator method, we show that DD, AD, and ETEST perform poorly, with neither MHA manufacturer nor specimen source significantly impacting method performance. Based on our data, we recommend that routine AST should not be performed for BCC isolates. If a provider requests AST, clinical microbiology laboratories should perform Clinical and Laboratory Standards Institute reference methodology for BMD (stored frozen) and report MIC only.

**IMPORTANCE:**

Antimicrobial susceptibility testing for the *Burkholderia cepacia* complex (BCC) is often used to determine eligibility for lung transplant in people with cystic fibrosis. However, problems with method performance have been reported. Here, we systematically evaluate the performance of reference broth microdilution, disk diffusion, agar dilution, and gradient diffusion (ETEST) for BCC organisms isolated from people with and without cystic fibrosis. We show that broth microdilution reproducibility is acceptable for levofloxacin, meropenem, minocycline, and trimethoprim-sulfamethoxazole, while ceftazidime was just below the acceptability cut-off. Regardless of specimen source, the results from disk diffusion, agar dilution, and ETEST do not correlate with broth microdilution. Based on these findings, we recommend that antimicrobial susceptibility testing should not be routinely performed for BCC, and if requested by the provider, only broth microdilution following Clinical and Laboratory Standards Institute guidelines should be used. Providers should be aware of the significant limitations of antimicrobial susceptibility testing methods for BCC.

## INTRODUCTION

The *Burkholderia cepacia* complex (BCC) includes 24 species of Gram-negative bacteria that cause opportunistic human infections (1, 2). While BCC is perhaps most well-known for causing respiratory infections in people with cystic fibrosis (CF) (3), BCC can also cause a variety of infections in people without CF, including respiratory infections (4) and bloodstream infections (5, 6), and has been associated with outbreaks due to contaminated medical devices and pharmaceutical products (7–11). The most common BCC species isolated from people with CF are *Burkholderia cenocepacia* and *Burkholderia multivorans* (12). Although *Burkholderia gladioli* is also commonly isolated from people with CF, it is not a member of BCC (12).

BCC infections are often difficult to treat due to both intrinsic and acquired resistance (13–15), problems with the performance of antimicrobial susceptibility testing (AST) methods, and no data correlating AST with clinical outcomes (16). BCC is intrinsically resistant to many antibiotics, including aminoglycosides and polymyxins (17), leaving clinicians with few options for treatment. As of this writing, the Clinical and Laboratory Standards Institute (CLSI) has published minimal inhibitory concentration (MIC) breakpoints for ceftazidime (CAZ), meropenem (MEM), minocycline (MIN), levofloxacin (LVX), trimethoprim-sulfamethoxazole (TMP-SMX), chloramphenicol (CAM), and ticarcillin-clavulanate (TIC) (17), with CAM being rarely clinically used and TIC not available in the USA. BCC can readily acquire resistance to these antimicrobials via a variety of mechanisms, making multi-drug resistance a problem (13, 15, 18). While CLSI disk diffusion (DD) and MIC breakpoints have been historically published for BCC, we and others have demonstrated variable performance for AST methods (19–21). In a European study of 155 BCC isolates from people with CF, Wootton et al. demonstrated low reproducibility of reference broth microdilution (BMD) (70.3%–84.5%) and poor correlation between agar dilution (AD), gradient diffusion (ETEST), and DD testing compared to BMD for CAZ, MEM, MIN, and TMP-SMX (21). In a study from Brazil, Fehlberg et al. evaluated the performance of BMD, AD, DD, and ETEST for testing CAZ, LVX, MEM, MIN, and TMP-SMX against 47 and 35 BCC isolates from people with and without CF, respectively, and found variable essential agreement (EA) and variable correlation between AST methods (19). In a previous study of 100 isolates collected from people with CF in North America, we showed acceptable BMD reproducibility for MEM and MIN (20) using a previously published cut-off of ≥95% (21); however, BMD reproducibility for CAZ, LVX, and TMP-SMX was more variable (82%–93%) (20). DD was poorly reproducible and did not correlate with BMD, with acceptance criteria met only for MEM (20). Based on the results from these previous studies, CLSI removed BCC DD breakpoints from the M100 ED34:2024 (17). Furthermore, the European Committee on Antimicrobial Susceptibility Testing (EUCAST) does not recommend routine AST to guide therapy for BCC because AST methods do not agree, nor do the results correlate with clinical outcomes (16, 22). Similarly, the Antimicrobial Resistance (AMR) in CF International Working Group and guidelines for clinical microbiology laboratories that process specimens from people with CF do not recommend routine AST due to limited studies correlating AST with clinical outcomes (23, 24). Perhaps, the most important consideration for evaluating the performance of BCC AST is that it is often used to determine eligibility for life-saving lung transplants in people with CF despite these issues (25, 26).

Previous studies had several limitations. First, our previous study evaluating BMD and DD was performed at a single site (20), and more robust data may be obtained by additional testing at a second site. Second, the performance of AST methods for isolates collected from people with and without CF has not been systematically evaluated. It is possible that AST methods may perform better for isolates from people without CF because they have not adapted to the CF lung environment (4, 15, 27–29). Third, our study and others evaluated Mueller-Hinton agar (MHA) from single manufacturers, and it has been shown for some other organisms that DD performance may vary depending on MHA brand (30, 31). Finally, our prior study did not evaluate other AST methods like AD or ETEST.

Here, we address the limitations of previous studies. We performed BMD for our original 100 BCC isolates collected from people with CF at a second site, evaluated the performance of BMD for 105 BCC isolates from people without CF, and analyzed DD performance using MHA from three manufacturers for all 205 BCC isolates. Finally, we evaluated the performance of AD and ETEST compared to BMD. Together, these data provide more insight into the performance of AST methods for BCC. Based on these data, CLSI is expected to remove BCC MIC breakpoints from the M100 ED35:2025 (32).

## MATERIALS AND METHODS

### Bacterial isolates and growth conditions

Two hundred five BCC isolates were evaluated in this study (Table 1; Table S1). One hundred isolates from persons with CF (CF isolates) were previously obtained from the Cystic Fibrosis Foundation *Burkholderia cepacia* Laboratory and Repository (BcLR) at the University of Michigan (*n* = 49 *B*. *cenocepacia* and *n* = 51 *B*. *multivorans*) (20). The purpose of the BcLR is to support the detection, prevention, and treatment of BCC infections in people with CF by accurately identifying BCC isolates, performing epidemiology studies, and maintaining an isolate repository that can be used to support research studies. All isolates were collected from CF sputum and represented the most recent isolate collected from chronically infected individuals. Isolates were previously identified to the species level by *recA* PCR and sequencing (33–35). One hundred five isolates from people without CF (non-CF isolates) were obtained from the University of British Columbia (UBC; *n* = 27), New York-Presbyterian/Columbia University Medical Center (NYP/CUMC; *n* = 12), and BcLR (*n* = 66) (Table 1). The non-CF isolates were identified to the species level as *B. cenocepacia* (*n* = 60), *B. multivorans* (*n* = 40), *Burkholderia cepacia* (*n* = 3), and *Burkholderia contaminans* (*n* = 2) via *recA* PCR and sequencing (*n* = 85) (33, 34) or whole genome sequencing (*n* = 20). Fifty-one and 54 non-CF isolates were from respiratory and non-respiratory sources, respectively (Table 2; Table S1). To maximize strain diversity and reduce the possibility of clonality, all isolates were collected from different individuals. Quality control (QC) strains used in this study include *Escherichia coli* ATCC 25922, *Klebsiella pneumoniae* ATCC 700603, *Pseudomonas aeruginosa* ATCC 27853, and *Staphylococcus aureus* ATCC 29213.

**TABLE 1:** BCC isolates analyzed in this study^*a*^

BCC species	Non-CF isolates			CF isolates	Total
	UBC	NYP/CUMC	BcLR	BcLR	
*B. cenocepacia*	19	6	35	49	109
*B. multivorans*	8	1	31	51	91
*B. cepacia*	0	3	0	0	3
*B. contaminans*	0	2	0	0	2
Total	27	12	66	100	205

^*a*^ BCC, *Burkholderia cepacia* complex; CF, cystic fibrosis; UBC, University of British Columbia; NYP/CUMC, New York-Presbyterian/Columbia University Medical Center; BcLR*, Burkholderia cepacia* Laboratory and Repository.

**TABLE 2:** Specimen sources, non-CF isolates^*a*^

Source	Number of isolates	Percentage of total isolates
Respiratory		
Bronchoalveolar lavage	10	10
Bronchus tissue	1	1
Endotracheal tube	4	4
Lung biopsy	1	1
Respiratory, not specified	1	1
Sinus	1	1
Sputum	23	22
Throat swab	1	1
Tracheal aspirate	6	6
Tracheal secretion	3	3
Sub-total	51	49
Non-respiratory		
Abdomen	1	1
Abscess	3	3
Ascites fluid	1	1
Blood	24	23
Body fluid	1	1
Bone	1	1
Colon tissue	1	1
Cerebrospinal fluid	1	1
Dialysate	1	1
Ear	2	2
Lymph node	1	1
Neck drainage	1	1
Synovial fluid	1	1
Urine	13	12
Wound	2	2
Sub-total	54	51
Grand total	105	100

^*a*^ CF, cystic fibrosis.

### BMD

BMD was performed at Vanderbilt University Medical Center (VUMC, Nashville, TN) following CLSI methods (36). The following antimicrobials were tested: CAZ, LVX, MEM, MIN, and TMP-SMX. Testing for non-CF isolates was performed from October 2021 to March 2022 using custom frozen reference BMD panels prepared by VUMC following CLSI methods (BD Difco). The following antimicrobial dilution ranges were used: 0.06 μg/mL–128 μg/mL, CAZ (Sigma); 0.03 μg/mL–64 μg/mL, LVX (Sigma), MEM (Sigma), and MIN (MedChemExpress); and 0.008/0.14 μg/mL–16/304 µg/mL, TMP-SMX (Sigma). For CF isolates, testing was performed from June 2022 to December 2022 using custom frozen reference BMD panels prepared by ThermoFisher with the same antimicrobial dilution ranges as listed above. Isolates were stored at −70°C. Frozen stocks were sub-cultured onto 5% sheep blood agar (SBAP) and incubated at 35°C in ambient air overnight or until visible colonies were observed. These isolates were sub-cultured onto 5% SBAP and incubated at 35°C in ambient air overnight for 20–24 hours. Isolated colonies were used to make a cell suspension with turbidity equivalent to a 0.5 McFarland standard. BMD panels were inoculated according to CLSI guidelines and incubated at 35°C in ambient air for 20–24 hours. MICs were read at 20–24 hours by two different operators, and disagreement was resolved by a third operator. All non-CF isolates (*n* = 105) were tested in triplicate on the same day using three separate 0.5 McFarland standards. Due to contamination issues thought to have been encountered during isolate shipment, a subset of CF isolates was tested in triplicate (*n* = 34 in triplicate, *n* = 1 in duplicate, *n* = 65 in single replicate, and two were discarded due to contamination, Table S1). Replicates were performed as described above. QC was performed each day of testing using *E. coli* ATCC 25922 and *P. aeruginosa* ATCC 27853.

### DD

DD was performed for all isolates at VUMC following CLSI methods. The following antimicrobials were tested: CAZ, LVX, MEM, MIN, and TMP-SMX (BD). DD and BMD were performed simultaneously using the same 0.5 McFarland standard for each isolate. MHA from BD, Remel, and Hardy Diagnostics (HD) were tested. The MHA lot numbers were the same within the isolate groups, CF and non-CF, but were different between the isolate groups. DD plates were incubated at 35°C in ambient air for 20–24 hours. Zones of inhibition were read by two different operators, and disagreement was resolved by a third operator. QC was performed each day of testing using *E. coli* ATCC 25922 and *P. aeruginosa* ATCC 27853.

### AD

AD was performed in single replicate at the Mayo Clinic (Rochester, MN) for all isolates following CLSI methods. The following antimicrobials were tested: CAZ, LVX, MEM, MIN, and TMP-SMX. Isolates were stored at −70°C. Frozen stocks were sub-cultured onto SBAP and incubated at 35°C in ambient air overnight or until visible colonies were observed. These isolates were sub-cultured onto 5% SBAP and incubated at 35°C in ambient air overnight for 20–24 hours. Isolated colonies were used to make a cell suspension with turbidity equivalent to a 0.5 McFarland standard. Custom agar plates prepared in-house were inoculated according to CLSI methods and incubated at 35°C in ambient air for 24 hours. MICs were read at 20–24 hours by a single operator. Four isolates were discarded due to contamination. QC was performed each day of testing using *E. coli* ATCC 25922, *K. pneumoniae* ATCC 700603, *P. aeruginosa* ATCC 27853, and *S. aureus* ATCC 29213.

### ETEST

ETEST (bioMérieux) was performed in single replicate for CAZ, LVX, MEM, MIN, and TMP-SMX at Harbor-UCLA Medical Center (Torrance, CA) for all isolates. While *Pseudomonas* spp., *Acinetobacter* spp., and *Stenotrophomonas maltophilia* are included in the ETEST indications for use, BCC is not for any of the antimicrobials tested. Therefore, the methods used were extrapolated from these other organisms. Isolates were stored at −70°C. Frozen stocks were sub-cultured onto SBAP and incubated at 35°C in ambient air overnight or until visible colonies were observed. These isolates were sub-cultured onto 5% SBAP and incubated at 35°C in ambient air overnight for 20–24 hours. Isolated colonies were used to make a cell suspension with turbidity equivalent to a 0.5 McFarland standard. ETEST was performed following the manufacturer’s instructions for aerobes (0.5 McFarland in 0.85% NaCl) on Remel MHA. MICs were read by single operators after 20–24 hours or 48 hours for slow-growing isolates per the manufacturer’s instructions for other non-fermenting Gram-negative organisms. Four isolates were discarded due to contamination. QC was performed each day of testing using *E. coli* ATCC 25922 and *P. aeruginosa* ATCC 27853.

### Data analysis

#### Breakpoints applied in this study

Breakpoints applied in this study were interpreted using the CLSI M100 Performance Standards for Antimicrobial Susceptibility Testing, 33rd Edition, 2023 (37) (Table 3). Since no LVX DD breakpoints are currently published by CLSI or EUCAST for BCC, we generated LVX DD breakpoints using disk-to-MIC correlates on the diffusion Breakpoint Testing Estimation Software (dBETS; version 1.5) (38, 39). The mode or median (for isolates with no mode) MIC and corresponding mode DD zone size measured on BD, Remel, and HD MHA were used for data input (*n* = 203 BCC isolates). dBETS generated the following BCC LVX DD breakpoints: ≥19 mm, susceptible (S); 17 mm–18 mm intermediate (I); and ≤16 mm, resistant (R) (Table 3).

**TABLE 3:** Breakpoints applied in this study^*a*^

Antimicrobial agent	Interpretive categories and zone diameter breakpoints (mm)^*b*^			Interpretive categories and MIC breakpoints (µg/mL)^*b*^		
	S	I	R	S	I	R
CAZ	≥21	18–20	≤17	≤8	16	≥32
LVX^*c*^	≥19^*c*^	17–18^*c*^	≤16^*c*^	≤2	4	≥8
MEM	≥20	16–19	≤15	≤4	8	≥16
MIN	≥19	15–18	≤14	≤4	8	≥16
TMP-SMX	≥16	11–15	≤10	≤2/38	–	≥4/76

^*a*^ CAZ, ceftazidime; MEM, meropenem; MIN, minocycline; TMP-SMX, trimethoprim-sulfamethoxazole; –, not applicable.

^*b*^ All DD and MIC breakpoints applied in this study are from CLSI M100 ED33:2023 except DD breakpoints for LVX.

^*c*^ LVX DD breakpoints were established using dBETS as described in Materials and Methods.

#### BMD reproducibility

BMD reproducibly was defined as the number of isolates with MICs the same as or within ±1 log_2_ dilution between replicates. Acceptance criteria was ≥95% based on a previous BCC AST study (21). Only isolates with on-scale MICs were included in the reproducibility analyses. Three BMD reproducibility analyses were performed: (i) all BCC isolates (*n* = 139 in triplicate); (ii) CF isolates (*n* = 34 in triplicate); and (iii) non-CF isolates (*n* = 105 in triplicate).

#### Comparison of DD to BMD

DD performed on each MHA brand was compared to BMD as the gold standard. The mode or median MIC (*n* = 34 in triplicate) or single replicate MIC (*n* = 169) was utilized. The error rate-bounded method was used to calculate very major errors (VME), major errors (ME), and minor errors (MI) per CLSI M23 ED6:2023 (40). Acceptance criteria for VME, ME, and MI are shown at the bottom of Tables 5 to 7 and 9. Analyses were performed for all isolates (*n* = 203), CF isolates (*n* = 98), non-CF isolates (*n* = 105), and non-CF isolates from respiratory (*n* = 51) and non-respiratory sources (*n* = 54). The number of errors between MHA brands, CF and non-CF isolates, and non-CF isolates from respiratory and non-respiratory sources was evaluated by χ^2^ tests using GraphPad Prism software (version 10) with *P*-value <0.05 considered significant.

#### Composite BMD MIC

Since AD and ETEST were performed at separate sites from DD and BMD, a composite BMD MIC was used as the comparator. The composite MIC was calculated using data for non-CF isolates tested at VUMC and CF isolates tested at both VUMC and the Clinical Microbiology Institute where our previous study was performed (20). All off-scale MICs were truncated to the last observable value. After truncation, the mode or median MIC (for isolates with no mode) was calculated. The final number of BMD replicates between the two sites are as follows: non-CF isolates, *n* = 105 in triplicate; CF isolates, *n* = 63 isolates with four replicates; *n* = 1 isolate with five replicates; *n* = 34 isolates with six replicates.

#### Comparison of AD to composite BMD

EA (MIC within ±1 log_2_ dilution) was calculated between AD and composite BMD MIC for all isolates (*n* = 201). AD was compared to the composite BMD MIC using the error rate-bounded method as described above. We compared AD to the composite BMD MIC for all isolates, CF isolates, non-CF isolates, and non-CF isolates from respiratory and non-respiratory sources.

#### Comparison of ETEST to composite BMD

For ETEST, MICs from 24 hours (*n* = 130) or 48 hours (*n* = 71) were used depending on whether sufficient growth was observed. EA was calculated between ETEST and the composite BMD MIC for all isolates (*n* = 201). The error rate-bounded method was used to evaluate ETEST compared to the composite BMD MIC as described above. We compared ETEST to the composite BMD MIC for CF isolates, non-CF isolates, and non-CF isolates from respiratory and non-respiratory sources.

## RESULTS

### MIC distributions

**Fig 1:**
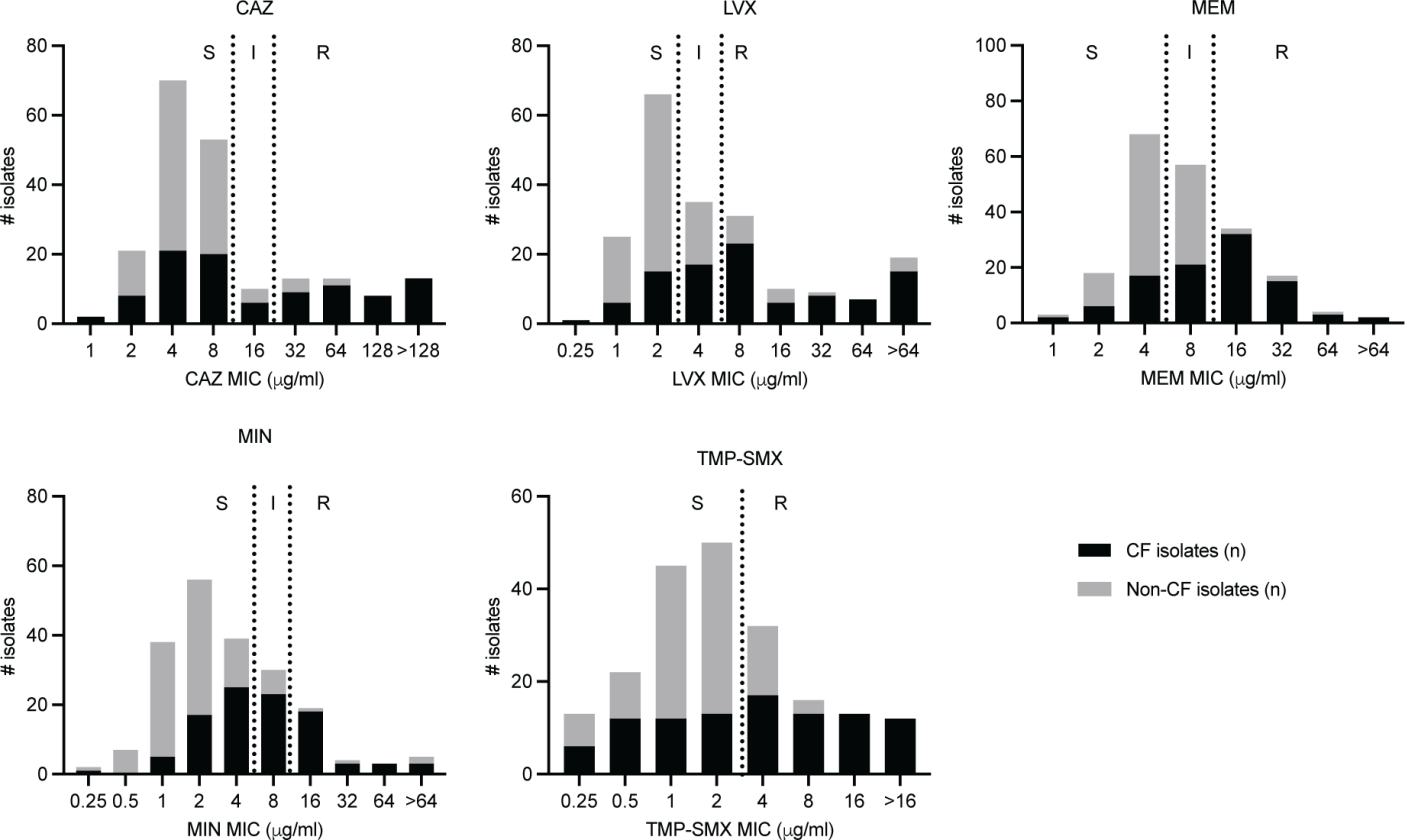
MIC distribution for BCC isolates tested against CAZ, LVX, MEM, MIN, and TMP-SMX as determined by BMD. Black dashed lines indicate BCC breakpoints from CLSI M100 ED33:2023. Black = CF isolates (*n* = 98) and gray = non-CF isolates (*n* = 105). #, number.

A total of 205 BCC isolates were included in this study (Table 1; Table S1). The isolate set included 100 CF isolates previously tested by DD and BMD at another site and 105 non-CF isolates from respiratory (*n* = 51) and non-respiratory (*n* = 54) sources (Table 2; Table S1). For non-CF isolates, blood (*n* = 24), sputum (*n* = 23), and urine (*n* = 13) were the most common sources. Isolates were tested using BMD against CAZ, LVX, MEM, MIN, and TMP-SMX, and MICs were interpreted using the breakpoints listed in Table 3. Data for two isolates were discarded due to contamination encountered during the testing process (*n* = 203). Susceptibility rates were highest for CAZ (72%), followed by MIN (70%), TMP-SMX (64%), LVX (45%), and MEM (44%) (Fig. 1). For each antimicrobial, CF isolates exhibited more resistance to all antimicrobials tested as compared to non-CF isolates: 42% and 6% were resistant to CAZ, 60% and 16% were resistant to LVX, 53% and 5% were resistant to MEM, 28% and 4% were resistant to MIN, and 56% and 18% were resistant to TMP-SMX, respectively.

### BMD reproducibility

BMD reproducibility was defined as ±1 log_2_ doubling dilution between replicates. When all BCC isolates were included in the analysis, reproducibility criteria were met for LVX, MEM, MIN, and TMP-SMX (≥95%); reproducibility for CAZ was just below the acceptance cut-off (93%) (Table 4). For CF isolates, reproducibility for CAZ was poor (81%), and while acceptance criteria for MEM were not met, reproducibility was very close to the ≥95% cut-off at 94%. Reproducibility for LVX, MIN, and TMP-SMX met acceptance criteria and ranged from 97 to 100% (Table 4). For non-CF isolates, reproducibility ranged from 96 to 100%, with acceptance criteria met for all antimicrobials (Table 4). Overall, except for CAZ, reproducibility of antimicrobials between CF and non-CF isolates was comparable (94%–100%).

**TABLE 4:** BMD reproducibility^*a*^

Antibiotic	All (*n* = 139)	CF (*n* = 34)	Non-CF (*n* = 105)
CAZ	**93%** (**126/136**)	**81%** (**25/31**)	96% (101/105)
LVX	98% (125/128)	100% (27/27)	97% (98/101)
MEM	99% (135/137)	**94%** (**30/32**)	100% (105/105)
MIN	98% (132/135)	100% (32/32)	97% (100/103)
TMP-SMX	96% (129/134)	97% (28/29)	96% (101/105)

^*a*^ *n*, the number of isolates tested in triplicate. Percentages indicate percentage of isolates with on-scale MICs meeting reproducibility criteria. Fractions in parentheses indicate numbers of isolates meeting reproducibility criteria. BMD reproducibility was defined as the number of isolates with MICs the same as or within ±1 log_2_ dilution between replicates. Acceptance criteria was ≥95%. Bold font indicates where acceptance criteria was not met.

### Performance of DD

LVX DD breakpoints are not currently published by CLSI or EUCAST. Previously, we applied *P. aeruginosa* LVX breakpoints to interpret BCC DD results. To overcome this limitation, we generated LVX DD breakpoints for BCC using dBETS (38) as described above. The BCC LVX DD breakpoints used in this study are shown in Table 3 and are ≥19 mm, susceptible, 17 mm–18 mm, intermediate, and ≤16 mm, resistant.

**Fig 2:**
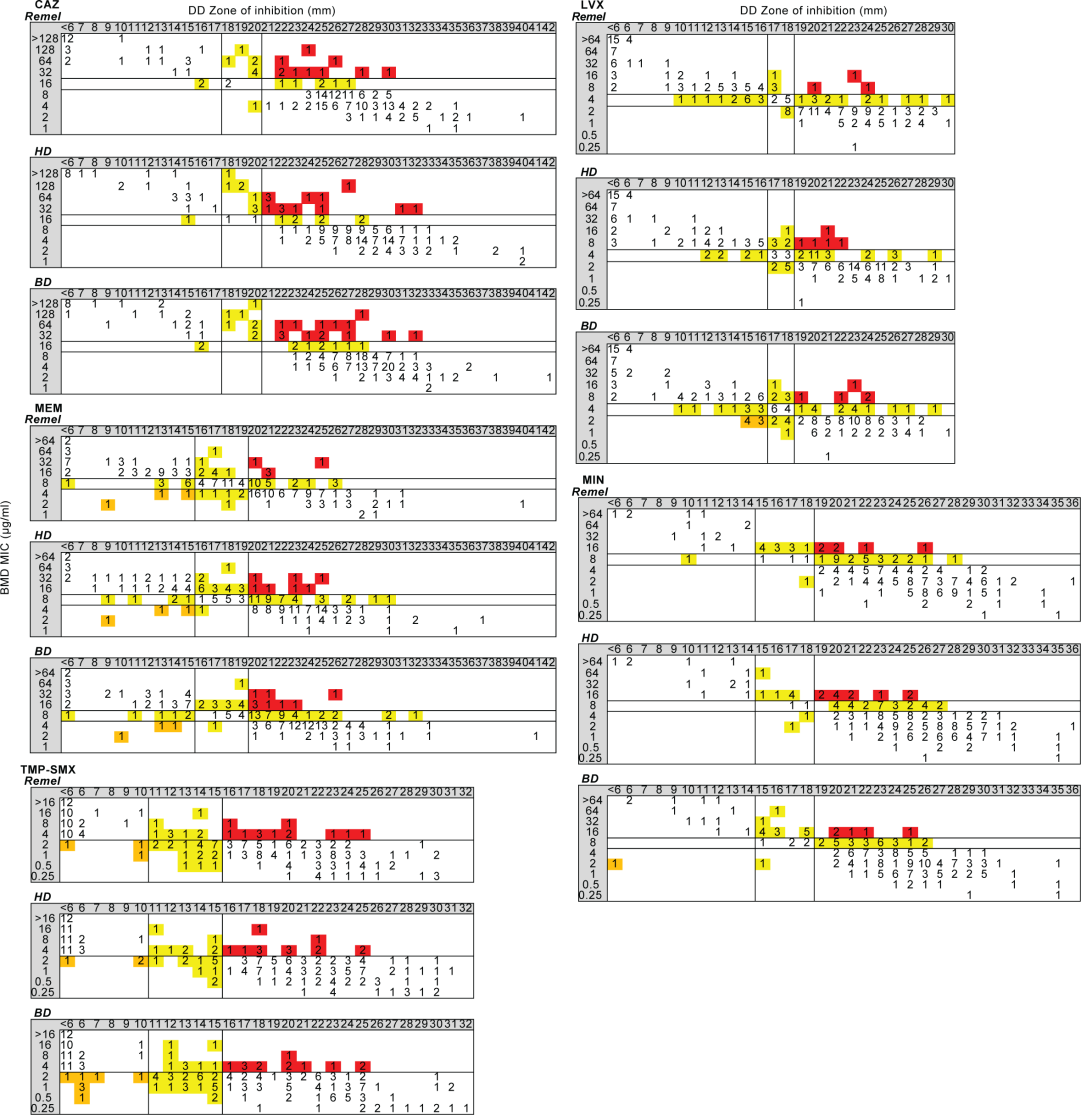
Scattergrams comparing DD on three different MHA brands to BMD for CAZ, LVX, MEM, MIN, and TMP-SMX, all BCC isolates. Scattergrams were generated by comparing the mode or median BMD MIC (y-axis) to DD zone diameter (x-axis) as measured on Remel, HD, or BD MHA. Solid lines within each scattergram indicate the breakpoints applied. Red, VME; orange, ME; and yellow, MI.

DD was performed using MHA from three different manufacturers: Remel, HD, and BD. For CF and non-CF isolates, seven isolates and one isolate exhibited faint growth, respectively, after incubation, but still met the CLSI standards for reading the zone of inhibition. DD was compared to BMD using the error rate-bounded method as described in CLSI M23 ED6:2023 (40). Analyses were performed for all isolates (Table 5; Fig. 2), CF isolates (Table 6; Fig. 4), non-CF isolates (Table 7; Fig. 5), and non-CF isolates from respiratory and non-respiratory sources (Tables S2 and S3; Fig. S1).

**TABLE 5:** Comparison of DD to BMD for three MHA brands, all BCC isolates (*n* = 203)^*a*^

Antibiotic	Type	Remel			HD			BD		
		VME	ME	MI	VME	ME	MI	VME	ME	MI
CAZ	≥I + 2	**8.8%** (**3/34**)	–	**11.8%** (**4/34**)	**17.6%** (**6/34**)	–	**14.7%** (**5/34**)	**17.6%** (**6/34**)	–	**17.6%** (**6/34**)
	I + 1 TO I − 1	9.2% (7/76)	0% (0/76)	15.8% (12/76)	**10.5%** (**8/76**)	0% (0/76)	14.5% (11/76)	**11.8%** (**9/76**)	0% (0/76)	15.8% (12/76)
	≤I − 2	–	0% (0/93)	1.1% (1/93)	–	0% (0/93)	0% (0/93)	–	0% (0/93)	0% (0/93)
LVX	≥I + 2	**2.2%** (**1/45**)	–	2.2% (1/45)	**2.2%** (**1/45**)	–	2.2% (1/45)	**2.2%** (**1/45**)	–	2.2% (1/45)
	I + 1 TO I − 1	1.5% (2/132)	0% (0/132)	29.5% (39/132)	3% (4/132)	0% (0/132)	31.1% (41/132)	3% (4/132)	5.3% (7/132)	27.3% (36/132)
	≤I − 2	–	0% (0/26)	0% (0/26)	–	0% (0/26)	0% (0/26)	–	0% (0/26)	3.8% (1/26)
MEM	≥I + 2	**8.7%** (**2/23**)	–	**8.7%** (**2/23**)	**13%** (**3/23**)	–	**13%** (**3/23**)	**13%** (**3/23**)	–	4.3% (1/23)
	I + 1 TO I − 1	1.9% (3/159)	1.3% (2/159)	27% (43/159)	2.5% (4/159)	1.3% (2/159)	37.7% (60/159)	3.8% (6/159)	1.3% (2/159)	37.7% (60/159)
	≤I − 2	–	**4.8%** (**1/21**)	4.8% (1/21)	–	4.8% (1/21)	0% (0/21)	–	**4.8%** (**1/21**)	0% (0/21)
MIN	≥I + 2	0% (0/12)	–	0% (0/12)	0% (0/12)	–	**8.3%** (**1/12**)	0% (0/12)	–	**16.7%** (**2/12**)
	I + 1 TO I − 1	6.8% (6/88)	0% (0/88)	**43.2%** (**38/88**)	**12.5%** (**11/88**)	0% (0/88)	39.8% (35/88)	5.7% (5/88)	0% (0/88)	**42%** (**37/88**)
	≤I − 2	–	0% (0/103)	1% (1/103)	–	0% (0/103)	1% (1/103)	–	1% (1/103)	1% (1/103)
TMP-SMX	≥R + 1	**4.9%** (**2/41**)	–	4.9% (2/41)	**4.9%** (**2/41**)	–	4.9% (2/41)	**2.4%** (**1/41**)	–	**7.3%** (**3/41**)
	R + S	**13.4%** (**11/82**)	2.4% (2/82)	28.0% (23/82)	**14.6%** (**12/82**)	3.7% (3/82)	18.3% (15/82)	**14.6%** (**12/82**)	4.9% (4/82)	28.0% (23/82)
	≤S − 1	–	1.3% (1/80)	**10%** (**8/80**)	–	0% (0/80)	**5%** (**4/80**)	–	**5%** (**4/80**)	**16.3%** (**13/80**)
Acceptance criteria	≥I + 2	<2%	ND	<5%	<2%	ND	<5%	<2%	ND	<5%
	I + 1 TO I − 1	<10%	<10%	<40%	<10%	<10%	<40%	<10%	<10%	<40%
	≤I − 2	ND	<2%	<5%	ND	<2%	<5%	ND	<2%	<5%

^*a*^ *n*, the number of isolates analyzed. Percentages indicate percentage of isolates with indicated errors, and fractions in parentheses indicate numbers of isolates with the indicated errors. Type indicates the MIC range of the isolates. ND, not determinable for the error type. Bold font indicates where acceptance criteria were not met. –, not determinable.

**TABLE 6:** Comparison of DD to BMD for three MHA brands, CF isolates (*n* = 98)^*a*^

Antibiotic	Type	Remel			HD			BD		
		VME	ME	MI	VME	ME	MI	VME	ME	MI
CAZ	≥I + 2	**9.4%** (**3/32**)	–	**12.5%** (**4/32**)	**18.8%** (**6/32**)	–	**15.6%** (**5/32**)	**18.8%** (**6/32**)	–	**18.8%** (**6/32**)
	I + 1 TO I − 1	**17.1%** (**6/35**)	0% (0/35)	20% (7/35)	**17.1%** (**6/35**)	0% (0/35)	20% (7/35)	**22.9%** (**8/35**)	0% (0/35)	20% (7/35)
	≤I − 2	–	0% (0/31)	0% (0/31)	–	0% (0/31)	0% (0/31)	–	0% (0/31)	0% (0/31)
LVX	≥I + 2	0% (0/36)	–	0% (0/36)	0% (0/36)	–	0% (0/36)	0% (0/36)	–	0% (0/36)
	I + 1 TO I − 1	0% (0/55)	0% (0/55)	30.9% (17/55)	3.6% (2/55)	0% (0/55)	34.5% (19/55)	3.6% (2/55)	0% (0/55)	29.1% (16/55)
	≤I − 2	–	0% (0/7)	0% (0/7)	–	0% (0/7)	0% (0/7)	–	0% (0/7)	0% (0/7)
MEM	≥I + 2	**5%** (**1/20**)	–	**5%** (**1/20**)	**10%** (**2/20**)	–	**10%** (**2/20**)	**10%** (**2/20**)	–	0% (0/20)
	I + 1 TO I − 1	4.3% (3/70)	0% (0/70)	30% (21/70)	5.7% (4/70)	0% (0/70)	**42.9%** (**30/70**)	7.1% (5/70)	0% (0/70)	**41.4%** (**29/70**)
	≤I − 2	–	**12.5%** (**1/8**)	0% (0/8)	–	**12.5%** (**1/8**)	0% (0/8)	–	**12.5%** (**1/8**)	0% (0/8)
MIN	≥I + 2	0% (0/9)	–	0% (0/9)	0% (0/9)	–	**11.1%** (**1/9**)	0% (0/9)	–	**11.1%** (**1/9**)
	I + 1 TO I − 1	9.1% (6/66)	0% (0/66)	**45.5%** (**30/66**)	**16.7%** (**11/66**)	0% (0/66)	39.4% (26/66)	7.6% (5/66)	0% (0/66)	**43.9%** (**29/66**)
	≤I − 2	–	0% (0/23)	4.3% (1/23)	–	0% (0/23)	4.3% (1/23)	–	0% (0/23)	4.3% (1/23)
TMP-SMX	≥R + 1	**2.6%** (**1/38**)	–	**5.3%** (**2/38**)	**2.6%** (**1/38**)	–	**5.3%** (**2/38**)	0% (0/38)	–	**7.9%** (**3/38**)
	R + S	**10%** (**3/30**)	6.7% (2/30)	33.3% (10/30)	**10%** (**3/30**)	6.7% (2/30)	20% (6/30)	**10%** (**3/30**)	**10%** (**3/30**)	23.3% (7/30)
	≤S − 1	–	**3.3%** (**1/30**)	**10%** (**3/30**)	–	0% (0/30)	**10%** (**3/30**)	–	0% (0/30)	**13.3%** (**4/30**)
Acceptance criteria	≥I + 2	<2%	ND	<5%	<2%	ND	<5%	<2%	ND	<5%
	I + 1 TO I − 1	<10%	<10%	<40%	<10%	<10%	<40%	<10%	<10%	<40%
	≤I − 2	ND	<2%	<5%	ND	<2%	<5%	ND	<2%	<5%

^*a*^ *n*, the number of isolates analyzed. Percentages indicate percentage of isolates with indicated errors, and fractions in parentheses indicate numbers of isolates with the indicated errors. Type indicates the MIC range of the isolates. ND, not determinable for the error type. Bold font indicates where acceptance criteria were not met. –, not determinable.

**TABLE 7:** Comparison of DD to BMD for three MHA brands, non-CF isolates (*n* = 105)^*a*^

Antibiotic	Type	Remel			HD			BD		
		VME	ME	MI	VME	ME	MI	VME	ME	MI
CAZ	≥I + 2	0.0% (0/2)	–	0.0% (0/2)	0.0% (0/2)	–	0.0% (0/2)	0.0% (0/2)	–	0.0% (0/2)
	I + 1 TO I − 1	2.4% (1/41)	0.0% (0/41)	12.2% (5/41)	4.9% (2/41)	0.0% (0/41)	9.8% (4/41)	2.4% (1/41)	0.0% (0/41)	12.2% (5/41)
	≤I − 2	–	0.0% (0/62)	1.6% (1/62)	–	0.0% (0/62)	0.0% (0/62)	–	0.0% (0/62)	0.0% (0/62)
LVX	≥I + 2	**11.1%** (**1/9**)	–	**11.1%** (**1/9**)	**11.1%** (**1/9**)	–	**11.1%** (**1/9**)	**11.1%** (**1/9**)	–	**11.1%** (**1/9**)
	I + 1 TO I − 1	2.6% (2/77)	0% (0/77)	28.6% (22/77)	2.6% (2/77)	0% (0/77)	28.6% (22/77)	2.6% (2/77)	9.1% (7/77)	26.0% (20/77)
	≤I − 2	–	0% (0/19)	0% (0/19)	–	0% (0/19)	0% (0/19)	–	0% (0/19)	**5.3%** (**1/19**)
MEM	≥I + 2	**33.3%** (**1/3**)	–	**33.3%** (**1/3**)	**33.3%** (**1/3**)	–	**33.3%** (**1/3**)	**33.3%** (**1/3**)	–	**33.3%** (**1/3**)
	I + 1 TO I − 1	0.0% (0/89)	2.2% (2/89)	24.7% (22/89)	0.0% (0/89)	2.2% (2/89)	33.7% (30/89)	1.1% (1/89)	2.2% (2/89)	34.8% (31/89)
	≤I − 2	–	0.0% (0/13)	**7.7%** (**1/13**)	–	0.0% (0/13)	0.0% (0/13)	–	0.0% (0/13)	0.0% (0/13)
MIN	≥I + 2	0.0% (0/3)	–	0.0% (0/3)	0.0% (0/3)	–	0.0% (0/3)	0.0% (0/3)	–	**33.3%** (**1/3**)
	I + 1 TO I − 1	0.0% (0/22)	0.0% (0/22)	36.4% (8/22)	0.0% (0/22)	0.0% (0/22)	**40.9%** (**9/22**)	0.0% (0/22)	0.0% (0/22)	36.4% (8/22)
	≤I − 2	–	0.0% (0/80)	0.0% (0/80)	–	0.0% (0/80)	0.0% (0/80)	–	1.3% (1/80)	0.0% (0/80)
TMP-SMX	≥R + 1	**33.3%** (**1/3**)	–	0.0% (0/3)	**33.3%** (**1/3**)	–	0.0% (0/3)	**33.3%** (**1/3**)	–	0.0% (0/3)
	R + S	**15.4%** (**8/52**)	0.0% (0/52)	25.0% (13/52)	**17.3%** (**9/52**)	1.9% (1/52)	17.3% (9/52)	**17.3%** (**9/52**)	1.9% (1/52)	30.8% (16/52)
	≤S − 1	–	0.0% (0/50)	**10.0%** (**5/50**)	–	0.0% (0/50)	2.0% (1/50)	–	**8.0%** (**4/50**)	**18.0%** (**9/50**)
Acceptance criteria	≥I + 2	<2%	ND	<5%	<2%	ND	<5%	<2%	ND	<5%
	I + 1 TO I − 1	<10%	<10%	<40%	<10%	<10%	<40%	<10%	<10%	<40%
	≤I − 2	ND	<2%	<5%	ND	<2%	<5%	ND	<2%	<5%

^*a*^ *n*, the number of isolates analyzed. Percentages indicate percentage of isolates with indicated errors and fractions in parentheses indicate numbers of isolates with the indicated errors. Type indicates the MIC range of the isolates. ND, not determinable for the error type. Bold font indicates where acceptance criteria were not met. –, not determinable.

**Fig 3:**
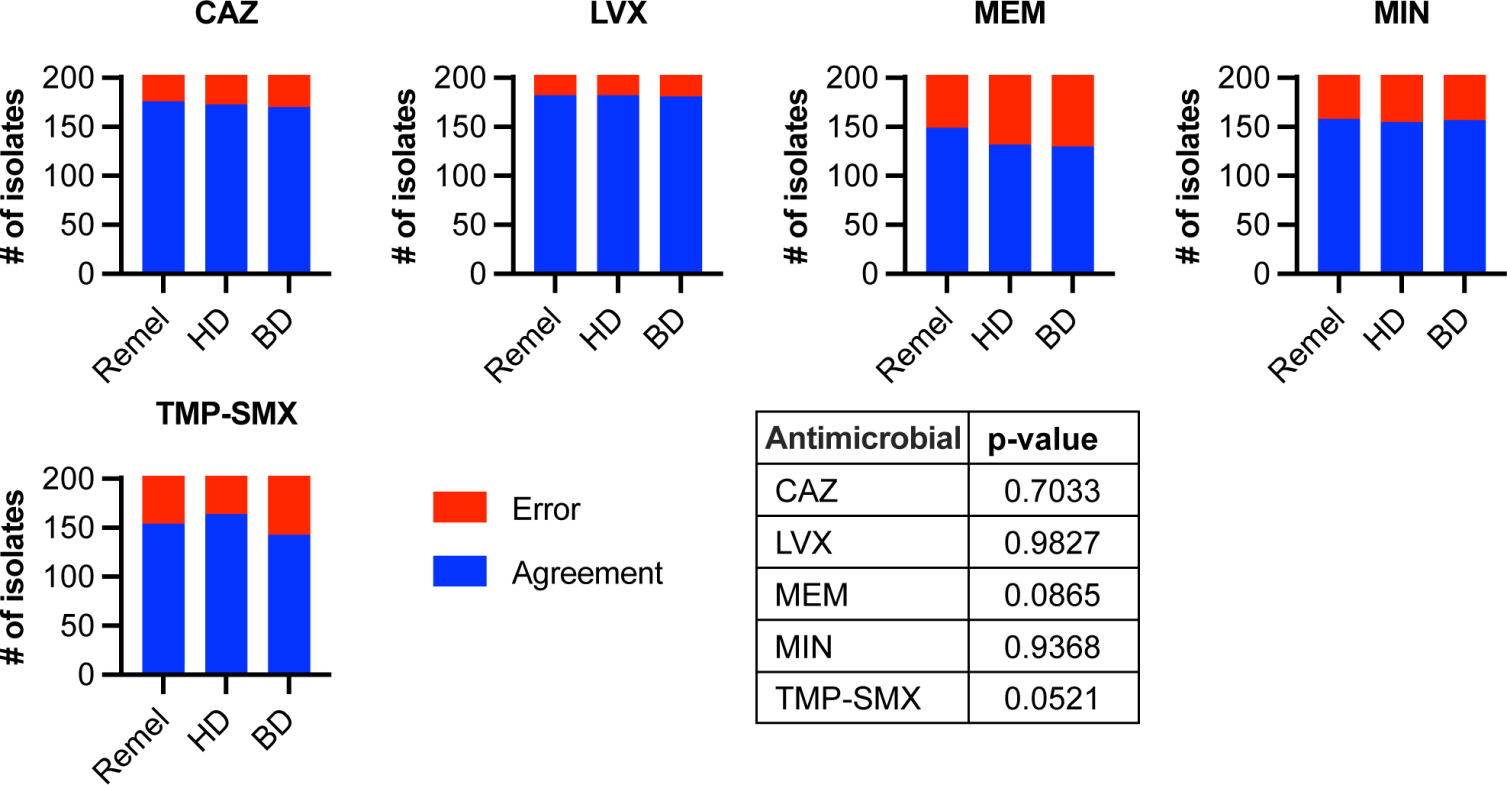
Comparison of error rates for CAZ, LVX, MEM, MIN, and TMP-SMX DD as compared to BMD, all BCC isolates. VMEs, MEs, and MIs for DD compared to BMD were summed for assays performed on three MHA brands (Remel, HD, and BD). The table summarizes χ^2^ analyses for each antimicrobial, testing whether errors are under- or over-represented for DD performed on three different MHA brands. *P*-value <0.05 was considered significant. Blue, agreement between methods; red, error between methods. #, number.

When all BCC isolates were included in the analyses (*n* = 203), DD failed to meet acceptance criteria for any antimicrobial using any MHA brand (Table 5; Fig. 2). Acceptance criteria for CAZ were not met due to unacceptable VME and MI rates using all MHA brands. For LVX, acceptance criteria were not met due to one VME in the ≥I + 2 category (1/45, 2.2%) on all MHA brands. VME, ME, and MI rates were unacceptable for MEM using all MHA brands, except MI rates were acceptable using BD MHA. For MIN, MI rates were unacceptable using all MHA brands, and VME rates were also unacceptable using HD MHA. Acceptance criteria were not met for TMP-SMX due to unacceptable VME and MI rates using all MHA brands, in addition to unacceptable ME rates using BD MHA. Notably, when we compared the number of VME, ME, and MI rates across MHA brands for CAZ, LVX, MEM, MIN, and TMP-SMX, there were no significant differences, indicating that DD performance is not impacted by MHA brand for BCC (Fig. 3).

**Fig 4:**
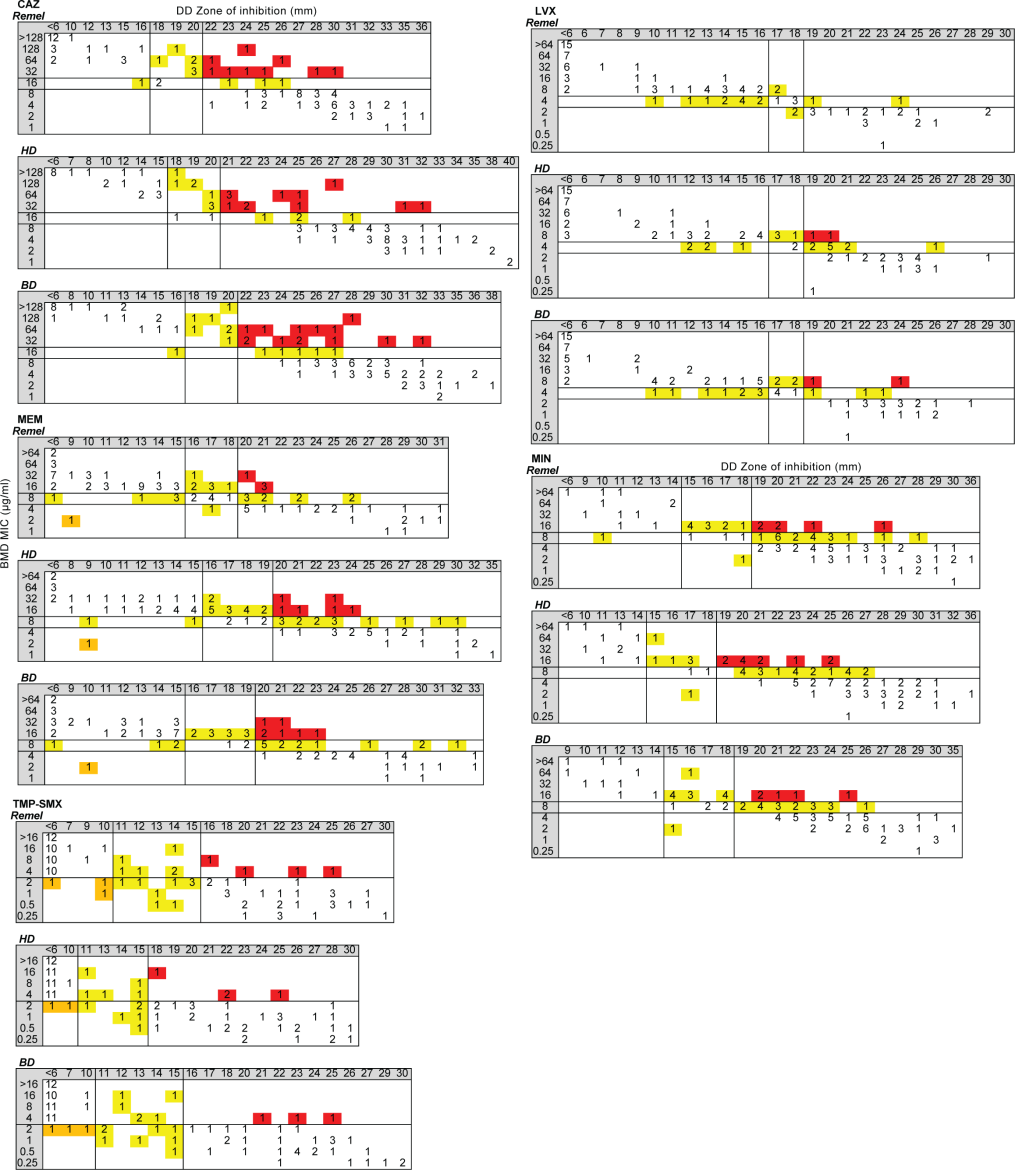
Scattergrams comparing DD on three different MHA brands to BMD for CAZ, LVX, MEM, MIN, and TMP-SMX, CF isolates. Scattergrams were generated by comparing the mode or median BMD MIC (y-axis) to DD zone diameter (x-axis) as measured on Remel, HD, or BD MHA for CF isolates. Solid lines within each scattergram indicate the breakpoints applied. Red, VME; orange, ME; and yellow, MI.

When only CF isolates were included in the DD to BMD analyses (*n* = 98), acceptance criteria were only met for LVX using MHA from all brands (Table 6; Fig. 4). For CAZ, VME and MI rates were unacceptable using all MHA brands. Acceptance criteria were not met for MEM due to unacceptable VME, ME, and MI rates using all MHA brands. For MIN, there were unacceptable MI rates on all MHA brands, in addition to unacceptable VME rates on HD MHA. Finally, acceptance criteria were not met for TMP-SMX due to unacceptable VME, ME, and/or MI rates on all MHA brands.

**Fig 5:**
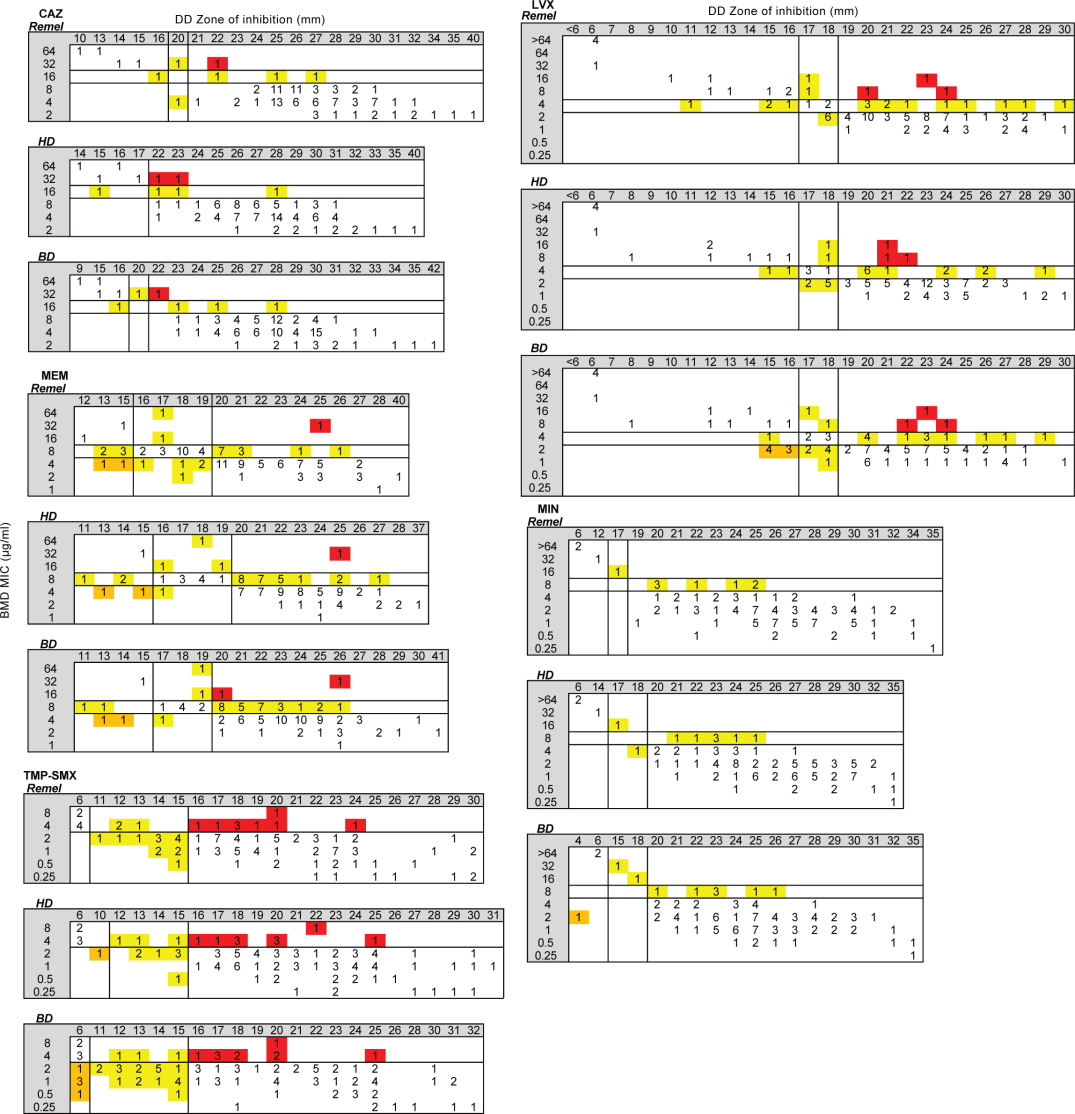
Scattergrams DD on three different MHA brands to BMD for CAZ, LVX, MEM, MIN, and TMP-SMX, non-CF isolates. Scattergrams were generated by comparing the mode or median BMD MIC (y-axis) to DD zone diameter (x-axis) as measured on Remel, HD, or BD MHA for non-CF isolates. Solid lines within each scattergram indicate the breakpoints applied. Red, VME; orange, ME; and yellow, MI.

For DD performance of non-CF isolates (*n* = 105), acceptance criteria were met for CAZ using all MHA brands and for MIN using Remel MHA (Table 7; Fig. 5). Using HD and BD MHA, MI rates were unacceptable for MIN. Acceptance criteria were not met for LVX due to one VME in the ≥I + 2 category (1/9, 11.1%) on all MHA brands and one MI using BD MHA (1/19, 5.3%). For MEM, VME and MI rates were unacceptable on all MHA brands. Finally, VME, ME, and/or MI rates were unacceptable for TMP-SMX on all MHA brands. Overall, non-CF isolates had fewer errors than CF isolates, but most antimicrobials still failed to meet acceptance criteria using MHA from all brands.

**Fig 6:**
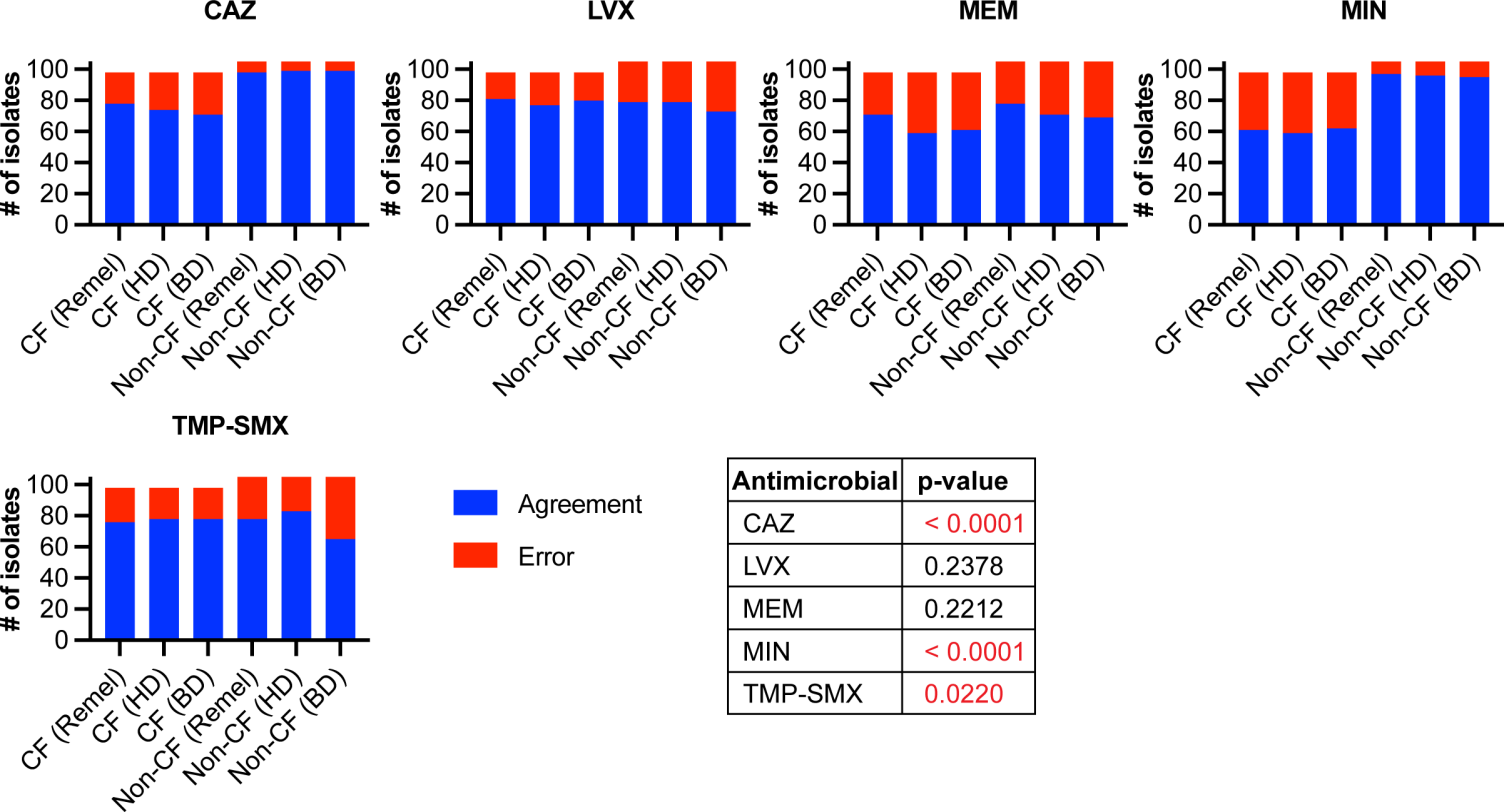
Comparison of error rates for CAZ, LVX, MEM, MIN, and TMP-SMX DD as compared to BMD, CF vs non-CF isolates. VMEs, MEs, and MIs for DD compared to BMD were summed for assays performed on three MHA brands (Remel, HD, and BD). The table summarizes χ^2^ analyses for each antimicrobial, testing whether errors are under- or over-represented for DD performed on three different MHA brands. *P*-value <0.05 was considered significant, and values <0.05 are highlighted red in the table. Blue, agreement between methods; red, error between methods. #, number.

To determine whether there were significant differences in DD performance between CF and non-CF isolates, VME, ME, and MI rates were compared for CAZ, LVX, MEM, MIN, and TMP-SMX between both isolate sets. Significantly more errors were seen with CAZ and MIN DD testing of CF isolates as compared to non-CF isolates, but, interestingly, non-CF isolates had significantly more errors than CF isolates for DD testing of TMP-SMX (Fig. 6). There were no significant differences between CF and non-CF isolates for DD testing of MEM and LVX (Fig. 6). These data indicate that DD performance is not impacted by specimen source, i.e., whether the isolate was collected from a person with or without CF.

We also compared non-CF isolates from respiratory (*n* = 51) and non-respiratory sources (*n* = 54) to further determine whether DD performance could be impacted by specimen source. DD performance was more variable for isolates from respiratory sources as compared to non-respiratory isolates. For non-CF isolates from respiratory sources (Table S1; Fig. S1), acceptance criteria were met for CAZ using Remel and BD MHA, but VME rates were unacceptable using HD MHA. Acceptance criteria for LVX were met using Remel and HD MHA, but ME and MI rates were unacceptable using BD MHA. For MEM, acceptance criteria were not met due to unacceptable VME and MI rates on all MHA brands. Acceptance criteria were not met for MIN due to unacceptable MI rates using Remel and HD MHA, while ME and MI rates were unacceptable on BD MHA. For TMP-SMX, acceptance criteria were not met using any MHA brand due to unacceptable VME, ME, and/or MI rates. For non-CF isolates from non-respiratory sources (Table S3; Fig. S1), acceptance criteria were met for CAZ and MEM using all MHA brands and for MIN using Remel and HD MHA. However, for MIN, MI rates were unacceptable using BD MHA due to 1 MI in the ≥I + 2 category (1/2; 50%). Acceptance criteria for LVX were not met due to unacceptable VME and MI rates using all MHA brands. Acceptance criteria for TMP-SMX were not met using any MHA brand due to unacceptable VME, ME, and/or MI rates. Despite the variability of DD performance based on specimen source for non-CF isolates, error rates were significantly higher only for TMP-SMX DD for non-CF isolates from respiratory sources, with the highest overall error rates using BD MHA (*P* = 0.0180, χ^2^ test, Fig. S2).

### Performance of AD

**Fig 7:**
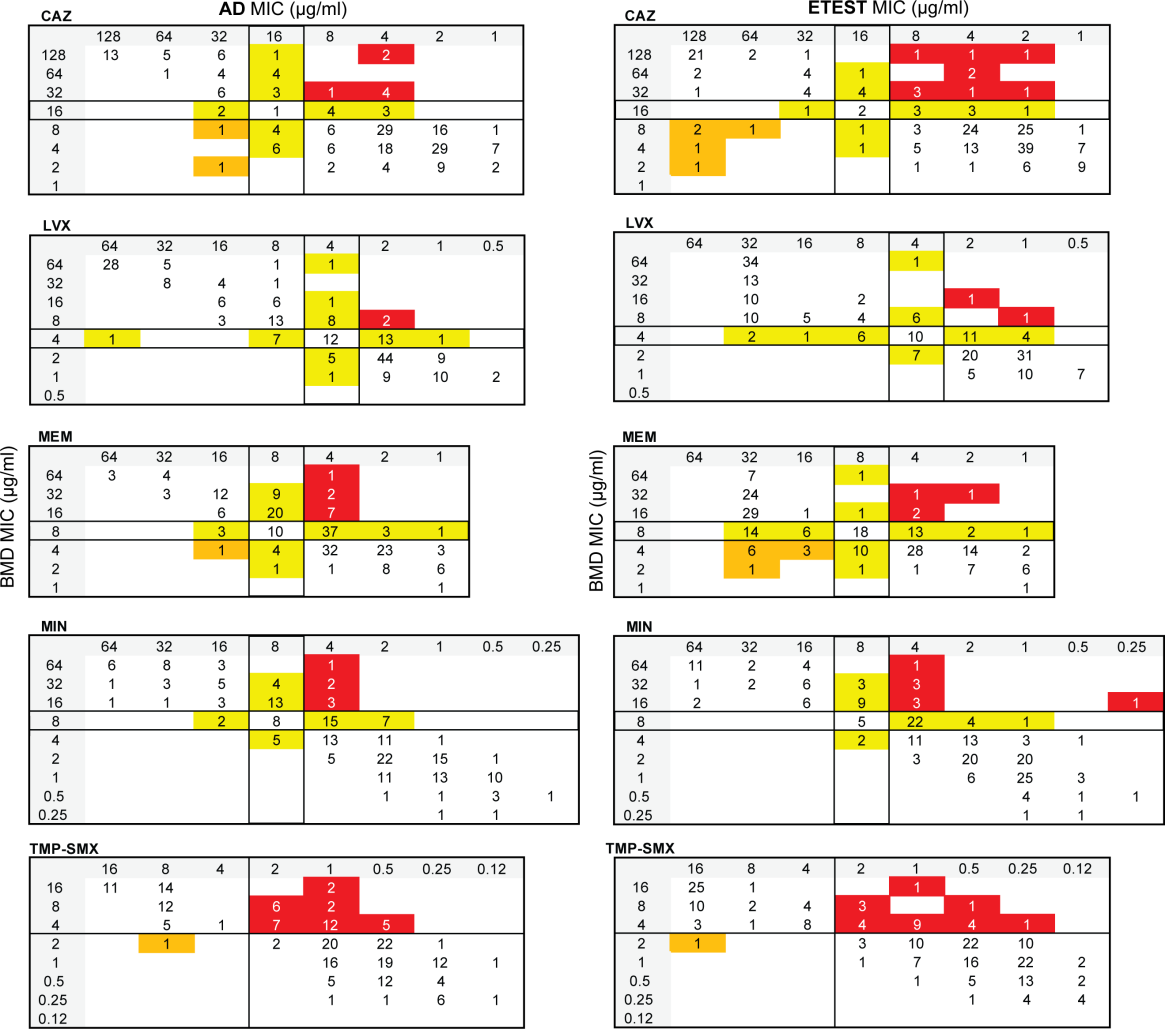
Scattergrams comparing AD (left) and ETEST (right) to BMD for CAZ, LVX, MEM, MIN, and TMP-SMX, all BCC isolates. Scattergrams were generated by comparing the composite BMD MIC (y-axis) to AD or ETEST MIC (x-axis). Solid lines within each scattergram indicate the breakpoints applied. Red, VME; orange, ME; and yellow, MI.

EA between AD and BMD was poor, with acceptance criteria being met only for LVX (96%). EA ranged from 68 to 88% for MIN (88%), MEM (86%), CAZ (73%), and TMP-SMX (68%) (Table 8). When AD was compared to BMD for all isolates, acceptance criteria were met only for LVX; VME and/or MI rates were unacceptable for CAZ, MEM, MIN, and TMP-SMX (Table 9; Fig. 7). For CF isolates, no antimicrobials met acceptance criteria, primarily due to unacceptable VME and MI rates (Table S4; Fig. S3). AD for non-CF isolates had fewer errors and therefore showed slightly better performance, with acceptance criteria being met for MIN (Table S4; Fig. S3). Acceptance criteria were not met for CAZ, LVX, MEM, and TMP-SMX due to unacceptable VME and/or MI rates. When comparing non-CF isolates from respiratory and non-respiratory sources (Table S5; Fig. S4), acceptance criteria were met for MIN and CAZ for respiratory and non-respiratory isolates, respectively. All other antimicrobials failed to meet acceptance criteria due to unacceptable VME and/or MI rates.

**TABLE 8:** EA of AD and ETEST with BMD, all BCC isolates (*n* = 201)^*a*^

Antibiotic	AD	ETEST
CAZ	**73%** (**146/201**)	**71%** (**143/201**)
LVX	96% (192/201)	**90%** (**180/201**)
MEM	**86%** (**173/201**)	**83%** (**166/201**)
MIN	**88%** (**176/201**)	**84%** (**168/201**)
TMP-SMX	**68%** (**136/201**)	**55%** (**110/201**)

^*a*^ Percentages indicate percentage of isolates with AD or ETEST MICs within ±1 log_2_ dilution of the BMD MIC. Fractions in parentheses in numbers of isolates with AD or ETEST MICs within ±1 log_2_ dilution of the BMD MIC. *n*, the number of isolates analyzed. Bold font indicates where acceptance criteria (EA ≥ 95%) were not met.

**TABLE 9:** Comparison of AD and ETEST to BMD, all BCC isolates (*n* = 201)^*a*^

Antibiotic	Type	AD			ETEST		
		VME	ME	MI	VME	ME	MI
CAZ	≥I + 2	**5.6%** (**2/36**)	–	**13.9%** (**5/36**)	**13.9%** (**5/36**)	–	2.8% (1/36)
	I + 1 TO I − 1	6.2% (5/81)	1.2% (1/81)	19.8% (16/81)	6.2% (5/81)	3.7% (3/81)	16% (13/81)
	≤I − 2	–	1.2% (1/84)	**7.1%** (**6/84**)	–	**2.4%** (**2/84**)	1.2% (1/84)
LVX	≥I + 2	0% (0/61)	–	3.3% (2/61)	1.6% (1/61)	–	1.6% (1/61)
	I + 1 TO I − 1	1.7% (2/118)	0% (0/118)	29.7% (35/118)	0.8% (1/118)	0% (0/118)	31.4% (37/118)
	≤I − 2	–	0% (0/22)	4.5% (1/22)	–	0% (0/22)	0% (0/22)
MEM	≥I + 2	**8.8%** (**3/34**)	–	**26.5%** (**9/34**)	**5.9%** (**2/34**)	–	2.9% (1/34)
	I + 1 TO I − 1	4.7% (7/150)	0.7% (1/150)	**45.3%** (**68/150**)	1.3% (2/150)	6% (9/150)	31.3% (47/150)
	≤I − 2	–	0% (0/17)	**5.9%** (**1/17**)	–	**5.9%** (**1/17**)	**5.9%** (**1/17**)
MIN	≥I + 2	**9.1%** (**3/33**)	–	**12.1%** (**4/33**)	**12.1%** (**4/33**)	–	**9.1%** (**3/33**)
	I + 1 TO I − 1	3.6% (3/83)	0% (0/83)	**50.6%** (**42/83**)	4.8% (4/83)	0% (0/83)	**45.8%** (**38/83**)
	≤I − 2	–	0% (0/85)	0% (0/85)	–	0% (0/85)	0% (0/85)
TMP-SMX	≥R + 1	**21.3%** (**10/47**)	–	NA	**10.6%** (**5/47**)	–	NA
	R + S	**31.6%** (**24/76**)	1.3% (1/76)	NA	**23.7%** (**18/76**)	1.3% (1/76)	NA
	≤S − 1	–	0% (0/78)	NA	–	0% (0/78)	NA
Acceptance criteria	≥I + 2	<2%	ND	<5%	<2%	ND	<5%
	I + 1 TO I − 1	<10%	<10%	<40%	<10%	<10%	<40%
	≤I − 2	ND	<2%	<5%	ND	<2%	<5%

^*a*^ *n*, the number of isolates analyzed. Percentages indicate percentage of isolates with indicated errors and fractions in parentheses indicate numbers of isolates with the indicated errors. Type indicates the MIC range of the isolates. ND, not determinable for the error type. Bold font indicates where acceptance criteria were not met. –, not determinable.

### Performance of ETEST

For ETEST, 36 isolates required 48 hours of incubation before reading the MIC because growth at 24 hours was too faint to read. The MICs of an additional 35 isolates were easier to read at 48 hours due to very light growth at 24 hours. Eight isolates had colonies within the zone of inhibition (CAZ, *n* = 3; LVX, *n* = 1; MEM, *n* = 3; MIN, *n* = 1), which were included in the MIC reading. Five isolates exhibited double zones of inhibition, and the zone with the higher MIC was recorded and used in the data analysis (CAZ, *n* = 3; MIN, *n* = 1; LVX, *n* = 1). EA with BMD ranged from 55 to 90%, with no antimicrobials meeting acceptance criteria; LVX had the highest EA with BMD (90%), followed by MIN (84%), MEM (83%), CAZ (71%), and TMP-SMX (55%) (Table 8). When ETEST was compared to BMD for all isolates, acceptance criteria were met for LVX, but VME, ME, and/or MI rates were unacceptable for CAZ, MEM, MIN, and TMP-SMX (Table 9; Fig. 7). Similarly, for CF isolates, acceptance criteria were met for LVX, but VME, ME, and/or MI rates were unacceptable for all other antimicrobials (Table S6; Fig. S5). ETEST of non-CF isolates performed slightly better, with acceptance criteria being met for CAZ and MIN, but VME and/or MI rates were unacceptable for LVX, MEM, and TMP-SMX (Table S6; Fig. S5). Among non-CF isolates from respiratory sources, acceptance criteria were met for MIN, but VME and/or MI rates were unacceptable for CAZ, LVX, MEM, and TMP-SMX (Table S7; Fig. S6). In contrast, ETEST performed better for non-CF isolates from non-respiratory isolates with acceptance criteria met for CAZ, MEM, and MIN, while VME rates were unacceptable for LVX and TMP-SMX (Table S7; Fig. S6).

## DISCUSSION

BCC organisms represent a unique challenge for clinicians, infection control practitioners, and clinical microbiologists because they can cause severe infections and outbreaks, are often multi-drug resistant, and present challenges with AST reproducibility and method agreement. Indeed, our BMD data reflect what is already appreciated about BCC: CF isolates tend to exhibit significantly more antimicrobial resistance than non-CF isolates. Our data for DD, AD, and ETEST performances demonstrate that AST methods for BCC perform poorly, and CF vs non-CF specimen source did not significantly impact results. Overall, providers should be made aware of the significant limitations for AST of BCC organisms, including lack of agreement between methods and data showing limited correlation with clinical outcomes.

Many clinical microbiology laboratories rely on DD for AST of rarely encountered organisms like BCC. We evaluated the performance of DD on MHA from Remel, HD, and BD compared to BMD, which overcame a limitation from our previous study where DD was performed using only Remel MHA (20). DD performed poorly compared to BMD regardless of MHA manufacturer for BCC isolates from people with CF and without CF. There was some variability between the performance with CF and non-CF isolates, and for non-CF isolates from respiratory and non-respiratory sources, but there was not a significant indication that differences among these specimen sources had a major impact on performance. Most concerning was the high number of VMEs, which results in false susceptibility. One limitation to our comparisons is that in the data for non-CF isolates, there were fewer resistant isolates because this population is often highly susceptible compared to CF isolates. Another limitation was that the data set used to generate LVX DD breakpoints was the same as that used to evaluate DD compared to BMD, which may introduce bias into the data. Regardless, the poor performance of DD compared to BMD shows that this method should not be used for BCC AST, which resulted in removal of DD breakpoints for BCC in M100 ED34:2024 (17), and lack of EUCAST DD breakpoints.

With the removal of BCC DD breakpoints from M100 ED34:2024, the question becomes whether MIC testing is reliable for BCC AST. Overall, BMD reproducibility was acceptable for LVX, MEM, MIN, and TMP-SMX, with CAZ just below the ≥95% acceptance cut-off (93%). Most antimicrobials were not impacted by CF vs non-CF specimen source except MEM, which was just below the acceptance cut-off for CF isolates (94%), and CAZ, for which reproducibility was the lowest for CF isolates (81%). These data are similar to our previous study of the same 100 CF isolates, where BMD reproducibility was lowest for CAZ (82%) and ranged from 88 to 97% for LVX (93%), MEM (97%), and MIN (97%), with TMP-SMX (88%) being the exception having lower reproducibility previously (20). However, while BMD may show acceptable or close to acceptable reproducibility results, MICs do not correlate with AD except for LVX. EA was poor for CAZ, MEM, MIN, and TMP-SMX (68-86%), and when compared to BMD, these antimicrobials did not meet acceptance criteria due to unacceptable VME and MI rates. Furthermore, we did not test AD reproducibility, so performance of this method beyond correlation with BMD is not known. These data raise questions as to which AST method provides the most clinical predictive measure of antimicrobial susceptibility for a BCC organism.

Without DD breakpoints for BCC, some clinical microbiology laboratories may use an alternative manual method like ETEST for BCC AST. Perhaps not surprisingly, ETEST performed poorly compared to BMD. EA was low for all antimicrobials tested (55%–84%) except LVX (90%), and only LVX met acceptance criteria when comparing ETEST to BMD. Like the results for DD and AD, specimen source did not significantly impact the results. It should be noted that BCC organisms are not included in the ETEST indications for use, which may be why the results were poor. We also noted that 71 isolates (35%) required 48-hour incubation to obtain interpretable MIC reads, although a comparison of 24-hour to 48-hour MIC reads was not performed.

Our study has several limitations. First, BMD for CF and non-CF isolates was performed on different dates using different BMD panels. For BMD reproducibility, fewer CF isolates were performed in triplicate compared to non-CF isolates (*n* = 34 and *n* = 105, respectively). However, the results are similar to our previous study where the same 100 CF isolates were tested in triplicate at another site (20). Additionally, AD and ETEST were not performed in parallel with BMD using the same inoculum, and only single replicates of both methods were performed. While ideally all AST methods would be performed from the same inoculum at the same laboratory, our results are highly similar to those of Wootton et al*.*, who also demonstrated low EA between AD and BMD (32.9%–80%) and ETEST and BMD (36.1%–83.9%) (21). When comparing CF to non-CF isolates and not aln-CF isolates from respiratory vs non-respiratory sources, there were fewer isolates within each category type for the error rate-bounded method, so one error may result in an antimicrobial not meeting acceptance criteria. Finally, we did not confirm inoculum sizes with corresponding plate counts, and we did not attempt to adjust the starting inoculum for BMD testing or other methods. Because inoculum effect has been shown to affect meropenem AST for other Gram-negative bacteria like *E. coli* (41)*,* future investigations into inoculum effects for BCC may offer opportunities to improve AST performance for these challenging organisms.

The results from this study and our previous study were presented to the CLSI AST Subcommittee in January 2024, when it was voted that all breakpoints should be removed for BCC (32). These changes will have a significant impact on clinical microbiology laboratories and providers, specifically those who treat people with CF and perform lung transplants. Several outstanding questions remain for BCC AST. First, in lieu of AST breakpoints, the CLSI AST Subcommittee has established tentative epidemiological cut-off values (ECVs) for CAZ, LVX, MEM, MIN, and TMP-SMX (H. K. Huse and P. A. Jorth., unpublished data, to be included in future M100 document). It remains to be seen how useful ECVs may be for guiding individual patient care (42). Second, due to the high antimicrobial resistance rates of CF isolates, it will be important to evaluate ECVs for newer antimicrobials like cefiderocol (43), ceftazidime-avibactam (44), piperacillin-avibactam (45), and meropenem-vaborbactam (46).

We recommend that clinical microbiology laboratories should not perform routine AST for BCC, which aligns with recommendations from EUCAST, the AMR in CF International Working Group (24), and recent guidelines for clinical microbiology laboratories that process specimens from people with CF (23). If AST is requested by the provider, reference BMD using frozen panels has demonstrated acceptable reproducibility and has the most robust data from our studies; however, it should be emphasized that AD does not correlate with BMD, and because no pharmacokinetic-pharmacodynamic (PK-PD) correlates are available, the optimal method for testing is not known. DD and ETEST are unreliable AST methods according to our data. With the removal of BCC breakpoints, we recommend that, if AST is performed upon clinician request, only MICs should be reported (i.e., without interpretations of susceptible, intermediate, or resistant) using frozen BMD panels following reference BMD CLSI methodology.

## Data Availability

Data supporting this study are available upon request.

## SUPPLEMENTAL MATERIAL

Supplemental material jcm.01480-24-s0001.docx Tables S2 to S7; Figures S1 to S6.

Table S1 jcm.01480-24-s0002.xlsx BCC strain information and AST results.

ASM does not own the copyrights to Supplemental Material that may be linked to, or accessed through, an article. The authors have granted ASM a non-exclusive, world-wide license to publish the Supplemental Material files. Please contact the corresponding author directly for reuse.

## References

[1] Parte AC, Sardà Carbasse J, Meier-Kolthoff JP, Reimer LC, Göker M. 2020. List of prokaryotic names with standing in nomenclature (LPSN) moves to the DSMZ. Int J Syst Evol Microbiol 70:5607–5612. doi:10.1099/ijsem.0.004332 32701423 PMC7723251

[2] Depoorter E, De Canck E, Peeters C, Wieme AD, Cnockaert M, Zlosnik JEA, LiPuma JJ, Coenye T, Vandamme P. 2020. Burkholderia cepacia complex taxon K: where to split? Front Microbiol 11:1594. doi:10.3389/fmicb.2020.01594 32760373 PMC7372133

[3] Somayaji R, Yau YCW, Tullis E, LiPuma JJ, Ratjen F, Waters V. 2020. Clinical outcomes associated with Burkholderia cepacia complex infection in patients with cystic fibrosis. Ann Am Thorac Soc 17:1542–1548. doi:10.1513/AnnalsATS.202003-204OC 32678679

[4] Shi H, Chen X, Chen L, Zhu B, Yan W, Ma X. 2023. Burkholderia cepacia infection in children without cystic fibrosis: a clinical analysis of 50 cases. Front Pediatr 11:1115877. doi:10.3389/fped.2023.1115877 37255574 PMC10225540

[5] El Chakhtoura NG, Saade E, Wilson BM, Perez F, Papp-Wallace KM, Bonomo RA. 2017. A 17-year nationwide study of Burkholderia cepacia complex bloodstream infections among patients in the United States Veterans Health Administration. Clin Infect Dis 65:1253–1259. doi:10.1093/cid/cix559 PMC5848224 29017247

[6] Luk KS, Tsang Y-M, Ho AY-M, To W-K, Wong BK-H, Wong MM-L, Wong Y-C. 2022. Invasive Burkholderia cepacia complex infections among persons who inject drugs, Hong Kong, China, 2016-2019. Emerg Infect Dis 28:323–330. doi:10.3201/eid2802.210945 34906288 PMC8798689

[7] Angrup A, Kanaujia R, Biswal M, Ray P. 2022. Systematic review of ultrasound gel associated Burkholderia cepacia complex outbreaks: clinical presentation, sources and control of outbreak. Am J Infect Control 50:1253–1257. doi:10.1016/j.ajic.2022.02.005 35158013

[8] Dolan SA, Dowell E, LiPuma JJ, Valdez S, Chan K, James JF. 2011. An outbreak of Burkholderia cepacia complex associated with intrinsically contaminated nasal spray. Infect Control Hosp Epidemiol 32:804–810. doi:10.1086/660876 21768765

[9] Du M, Song L, Wang Y, Suo J, Bai Y, Xing Y, Xie L, Liu B, Li L, Luo Y, Liu Y. 2021. Investigation and control of an outbreak of urinary tract infections caused by Burkholderia cepacian-contaminated anesthetic gel. Antimicrob Resist Infect Control 10:1. doi:10.1186/s13756-020-00855-x 33407871 PMC7789005

[10] Rhee C, Baker MA, Tucker R, Vaidya V, Holtzman M, Seethala RR, Bentain-Melanson M, Lenox J, Smith AR, Boyer JC, Gassett A, Brigl M, Sater M, Huntley M, Woolley AE, Goldberg HJ, Reilly K, Resnick A, Pearson M, Klompas M. 2022. Cluster of Burkholderia cepacia complex infections associated with extracorporeal membrane oxygenation water heater devices . Clin Infect Dis 75:1610–1617. doi:10.1093/cid/ciac200 35271726

[11] Sommerstein R, Führer U, Lo Priore E, Casanova C, Meinel DM, Seth-Smith HM, Kronenberg A, Koch D, Senn L, Widmer AF, Egli A, Marschall J, Anresis, Swissnoso. 2017. Burkholderia stabilis outbreak associated with contaminated commercially-available washing gloves, Switzerland, May 2015 to August 2016. Euro Surveill 22:17-00213. doi:10.2807/1560-7917.ES.2017.22.49.17-00213 PMC5727593 29233255

[12] Caverly L, Bumford A, Krot J, Kalikin L, Lipuma J. 2023. 629 updates in Burkholderia infection epidemiology in cystic fibrosis. J Cyst Fibros 22:S337. doi:10.1016/S1569-1993(23)01551-5

[13] Rhodes KA, Schweizer HP. 2016. Antibiotic resistance in Burkholderia species. Drug Resist Updat 28:82–90. doi:10.1016/j.drup.2016.07.003 27620956 PMC5022785

[14] Schaumburg F, Idelevich EA, Mellmann A, Kahl BC. 2022. Susceptibility of Burkholderia cepacia complex to ceftazidime/avibactam and standard drugs of treatment for cystic fibrosis patients. Microb Drug Resist 28:545–550. doi:10.1089/mdr.2021.0353 35512733

[15] Zhou J, Chen Y, Tabibi S, Alba L, Garber E, Saiman L. 2007. Antimicrobial susceptibility and synergy studies of Burkholderia cepacia complex isolated from patients with cystic fibrosis. Antimicrob Agents Chemother 51:1085–1088. doi:10.1128/AAC.00954-06 17158942 PMC1803131

[16] Lord R, Jones AM, Horsley A. 2020. Antibiotic treatment for Burkholderia cepacia complex in people with cystic fibrosis experiencing a pulmonary exacerbation. Cochrane Database Syst Rev 4:CD009529. doi:10.1002/14651858.CD009529.pub4 32239690 PMC7117566

[17] CLSI. 2024. Performance standards for antimicrobial susceptibility testing. 34th edition. Clinical and Laboratory Standards Institute.

[18] Becka SA, Zeiser ET, Barnes MD, Taracila MA, Nguyen K, Singh I, Sutton GG, LiPuma JJ, Fouts DE, Papp-Wallace KM. 2018. Characterization of the AmpC β-lactamase from Burkholderia multivorans. Antimicrob Agents Chemother 62:e01140-18. doi:10.1128/AAC.01140-18 30012762 PMC6153817

[19] Fehlberg LCC, Nicoletti AG, Ramos AC, Rodrigues-Costa F, de Matos AP, Girardello R, Marques EA, Gales AC. 2016. In vitro susceptibility of Burkholderia cepacia complex isolates: comparison of disk diffusion, Etest, agar dilution, and broth microdilution methods. Diagn Microbiol Infect Dis 86:422–427. doi:10.1016/j.diagmicrobio.2016.08.015 27638346

[20] Huse HK, Lee MJ, Wootton M, Sharp SE, Traczewski M, LiPuma JJ, Jorth P. 2021. Evaluation of antimicrobial susceptibility testing methods for Burkholderia cenocepacia and Burkholderia multivorans isolates from cystic fibrosis patients. J Clin Microbiol 59:e0144721. doi:10.1128/JCM.01447-21 34524889 PMC8601252

[21] Wootton M, Davies L, Pitman K, Howe RA. 2020. Evaluation of susceptibility testing methods for Burkholderia cepacia complex: a comparison of broth microdilution, agar dilution, gradient strip and EUCAST disc diffusion. Clin Microbiol Infect:S1198-743X(20)30708-4. doi:10.1016/j.cmi.2020.11.012 33253940

[22] EUCAST. 2013. Guidance document: antimicrobial susceptibility testing of Burkholderia cepacia complex (BCC). European Committee on Antimicrobial Susceptibility Testing.

[23] Saiman L, Waters V, LiPuma JJ, Hoffman LR, Alby K, Zhang SX, Yau YC, Downey DG, Sermet-Gaudelus I, Bouchara JP, Kidd TJ, Bell SC, Brown AW. 2024. Practical guidance for clinical microbiology laboratories: updated guidance for processing respiratory tract samples from people with cystic fibrosis. Clin Microbiol Rev 37:e0021521. doi:10.1128/cmr.00215-21 39158301 PMC11391703

[24] Somayaji R, Parkins MD, Shah A, Martiniano SL, Tunney MM, Kahle JS, Waters VJ, Elborn JS, Bell SC, Flume PA, VanDevanter DR, Antimicrobial Resistance in Cystic Fibrosis InternationalWorking Group. 2019. Antimicrobial susceptibility testing (AST) and associated clinical outcomes in individuals with cystic fibrosis: a systematic review. J Cyst Fibros 18:236–243. doi:10.1016/j.jcf.2019.01.008 30709744

[25] Alexander BD, Petzold EW, Reller LB, Palmer SM, Davis RD, Woods CW, Lipuma JJ. 2008. Survival after lung transplantation of cystic fibrosis patients infected with Burkholderia cepacia complex. Am J Transplant 8:1025–1030. doi:10.1111/j.1600-6143.2008.02186.x 18318775

[26] Aris RM, Routh JC, LiPuma JJ, Heath DG, Gilligan PH. 2001. Lung transplantation for cystic fibrosis patients with Burkholderia cepacia complex. Survival linked to genomovar type. Am J Respir Crit Care Med 164:2102–2106. doi:10.1164/ajrccm.164.11.2107022 11739142

[27] Leitão JH, Sousa SA, Cunha MV, Salgado MJ, Melo-Cristino J, Barreto MC, Sá-Correia I. 2008. Variation of the antimicrobial susceptibility profiles of Burkholderia cepacia complex clonal isolates obtained from chronically infected cystic fibrosis patients: a five-year survey in the major Portuguese treatment center. Eur J Clin Microbiol Infect Dis 27:1101–1111. doi:10.1007/s10096-008-0552-0 18600352

[28] Lieberman TD, Flett KB, Yelin I, Martin TR, McAdam AJ, Priebe GP, Kishony R. 2014. Genetic variation of a bacterial pathogen within individuals with cystic fibrosis provides a record of selective pressures. Nat Genet 46:82–87. doi:10.1038/ng.2848 24316980 PMC3979468

[29] Lieberman TD, Michel J-B, Aingaran M, Potter-Bynoe G, Roux D, Davis MR Jr, Skurnik D, Leiby N, LiPuma JJ, Goldberg JB, McAdam AJ, Priebe GP, Kishony R. 2011. Parallel bacterial evolution within multiple patients identifies candidate pathogenicity genes. Nat Genet 43:1275–1280. doi:10.1038/ng.997 22081229 PMC3245322

[30] Huse HK, Miller SA, Chandrasekaran S, Hindler JA, Lawhon SD, Bemis DA, Westblade LF, Humphries RM. 2018. Evaluation of oxacillin and cefoxitin disk diffusion and MIC breakpoints established by the clinical and laboratory standards institute for detection of mecA-mediated oxacillin resistance in Staphylococcus schleiferi. J Clin Microbiol 56:e01653-17. doi:10.1128/JCM.01653-17 29187565 PMC5786728

[31] Miller SA, Karichu J, Kohner P, Cole N, Hindler JA, Patel R, Richter S, Humphries RM. 2017. Multicenter evaluation of a modified cefoxitin disk diffusion method and PBP2a testing to predict mecA-mediated oxacillin resistance in atypical Staphylococcus aureus. J Clin Microbiol 55:485–494. doi:10.1128/JCM.02211-16 27903603 PMC5277518

[32] CLSI. To remove the Burkholderia cepacia complex breakpoints in M100. Clinical and Laboratory Standards Institute.

[33] Coenye T, Spilker T, Martin A, LiPuma JJ. 2002. Comparative assessment of genotyping methods for epidemiologic study of Burkholderia cepacia Genomovar III. J Clin Microbiol 40:3300–3307. doi:10.1128/JCM.40.9.3300-3307.2002 12202570 PMC130787

[34] Payne GW, Vandamme P, Morgan SH, Lipuma JJ, Coenye T, Weightman AJ, Jones TH, Mahenthiralingam E. 2005. Development of a recA gene-based identification approach for the entire Burkholderia genus. Appl Environ Microbiol 71:3917–3927. doi:10.1128/AEM.71.7.3917-3927.2005 16000805 PMC1169057

[35] Tabacchioni S, Ferri L, Manno G, Mentasti M, Cocchi P, Campana S, Ravenni N, Taccetti G, Dalmastri C, Chiarini L, Bevivino A, Fani R. 2008. Use of the gyrB gene to discriminate among species of the Burkholderia cepacia complex. FEMS Microbiol Lett 281:175–182. doi:10.1111/j.1574-6968.2008.01105.x 18312571

[36] CLSI. 2024. Methods for dilution antimicrobial susceptibility tests for bacteria that grow aerobically. Clinical and Laboratory Standards Institute.

[37] CLSI. 2023. Performance standards for antimicrobial susceptibility testing. 33rd ed. Clinical Laboratory Standards Institute.

[38] DePalma G, Turnidge J, Craig BA. 2017. Determination of disk diffusion susceptibility testing interpretive criteria using model-based analysis: development and implementation. Diagn Microbiol Infect Dis 87:143–149. doi:10.1016/j.diagmicrobio.2016.03.004 27856043

[39] Khan A, Pettaway C, Dien Bard J, Arias CA, Bhatti MM, Humphries RM. 2023. Evaluation of the performance of manual antimicrobial susceptibility testing methods and disk breakpoints for Stenotrophomonas maltophilia. Antimicrob Agents Chemother 95:e02631-20. doi:10.1128/aac.02631-20 33558287 PMC8092892

[40] CLSI. 2023. M23 Development of in vitro susceptibility testing criteria and quality control parameters. 6th ed. Clinical and Laboratory Standards Institute.

[41] Smith KP, Kirby JE. 2018. The inoculum effect in the era of multidrug resistance: minor differences in inoculum have dramatic effect on mic determination. Antimicrob Agents Chemother 62:e00433-18. doi:10.1128/AAC.00433-18 29784837 PMC6105823

[42] Sullivan KV, Long T, Hillesland E, Rhoads DD, Wojewoda CM, Zhang SX. 2024. Low utilization of epidemiological cutoff values to interpret in vitro antifungal susceptibility testing among clinical laboratory participants in two College of American Pathologists (CAP) proficiency testing programs. J Clin Microbiol 62:e0042124. doi:10.1128/jcm.00421-24 38920378 PMC11250375

[43] Nye C, Duckers J, Dhillon R. 2022. Cefiderocol to manage chronic, multi-drug-resistant Burkholderia cepacia complex infection in a patient with cystic fibrosis: a case report. Access Microbiol 4:acmi000413. doi:10.1099/acmi.0.000413 36415733 PMC9675169

[44] Spoletini G, Etherington C, Shaw N, Clifton IJ, Denton M, Whitaker P, Peckham DG. 2019. Use of ceftazidime/avibactam for the treatment of MDR Pseudomonas aeruginosa and Burkholderia cepacia complex infections in cystic fibrosis: a case series. J Antimicrob Chemother 74:1425–1429. doi:10.1093/jac/dky558 30649419

[45] Zeiser ET, Becka SA, Wilson BM, Barnes MD, LiPuma JJ, Papp-Wallace KM. 2019. “Switching Partners”: piperacillin-avibactam is a highly potent combination against multidrug-resistant Burkholderia cepacia complex and Burkholderia gladioli cystic fibrosis isolates. J Clin Microbiol 57:e00181-19. doi:10.1128/JCM.00181-19 31167848 PMC6663914

[46] Caverly LJ, Spilker T, Kalikin LM, Stillwell T, Young C, Huang DB, LiPuma JJ. 2019. In vitro activities of β-lactam-β-lactamase inhibitor antimicrobial agents against cystic fibrosis respiratory pathogens. Antimicrob Agents Chemother 64:e01595-19. doi:10.1128/AAC.01595-19 31611364 PMC7187596

