# Supplementary material for: Evaluation of antimicrobial susceptibility testing methods for Burkholderia cepacia complex isolates from people with and without cystic fibrosis
Source: J Clin Microbiol. 2025 Jan 22;63(2):e01480-24. doi: 10.1128/jcm.01480-24 (PMC11837569; doi:10.1128/jcm.01480-24)
Supplement: Supplemental material — Tables S2 to S7; Figures S1 to S6. [file jcm.01480-24-s0001.docx]

**Evaluation of antimicrobial susceptibility testing methods for *Burkholderia cepacia* complex isolates from people with and without cystic fibrosis**

Peter Jorth^1*^, Carmila Manuel^2^, Tracy McLemore^2^, Romney M. Humphries^2^, Nicolynn C. Cole^3^, Audrey N. Schuetz^3^, Dennis Garcia^4^, Maria Maldonado^4^, Natasha Rivero^4^, Anna Clara Milesi Galdino^1^, Diana Celedonio^1^, John J. LiPuma^5^, Daniel A. Green^6^, James E. A. Zlosnik^7^, Maria Traczewski^8^, Holly K. Huse^4*^

1. Department of Pathology and Laboratory Medicine, Department of Medicine, Department of Biomedical Sciences, Cedars Sinai Medical Center, Los Angeles, CA
2. Department of Pathology, Microbiology, & Immunology, Vanderbilt University Medical Center, Nashville, TN
3. Department of Laboratory Medicine and Pathology, Mayo Clinic, Rochester, MN
4. Department of Pathology, Harbor-UCLA Medical Center, Torrance, CA
5. Department of Pediatrics, University of Michigan, Ann Arbor, MI
6. Department of Pathology, New York Presbyterian/Columbia University Medical Center, New York, NY
7. Department of Pediatrics, University of British Columbia, Vancouver, BC
8. Clinical Microbiology Institute, Inc., Wilsonville, OR

*Correspondence: [hhuse@dhs.lacounty.gov](mailto:hhuse@dhs.lacounty.gov) or [peter.jorth@cshs.org](mailto:peter.jorth@cshs.org)

**Supplemental Information**

**Table S1. *Burkholderia cepacia* complex (BCC) strain information and AST results.**

BCC=*Burkholderia cepacia* complex. CF=cystic fibrosis. UBC=University of British Columbia. NYP/CUMC=New York-Presbyterian/Columbia University Medical Center. BcLR=*Burkholderia cepacia* Laboratory and Repository. Resp= respiratory isolate. Nonresp= non-respiratory isolate. CAN= Canada. USA= United States of America. CMI=Clinical Microbiology Institute. VUMC=Vanderbilt University Medical Center. AD=agar dilution. BMD=broth microdilution. CAZ=ceftazidime. LVX=levofloxacin. MEM=meropenem. MIN=minocycline. TMP-SMX=trimethoprim-sulfamethoxazole. HD=Hardy Diagnostics.

**Table S2. Comparison of DD to BMD for 3 MHA brands, non-CF isolates from respiratory sources (n=51).*^a^***

* a* DD=disk diffusion. BMD=broth microdilution. MHA=Mueller Hinton Agar. CF=cystic fibrosis. n=the number of isolates analyzed. Percentages indicate percentage of isolates with indicated errors and fractions in parentheses indicate numbers of isolates with the indicated errors. Type indicates the MIC range of the isolates. CAZ=ceftazidime. LVX=levofloxacin. MEM=meropenem. MIN=minocycline. TMP-SMX=trimethoprim-sulfamethoxazole. S=susceptible. I=intermediate. R=resistant. HD=Hardy Diagnostics. VME=very major error. ME=major error. MI=minor error. ND=not determinable for the error type. Red font indicates where acceptance criteria were not met.

**Table S3. Comparison of DD to BMD for 3 MHA brands, non-CF isolates from non-respiratory sources (n=54).*^a^***

* a* DD=disk diffusion. BMD=broth microdilution. MHA=Mueller Hinton Agar. CF=cystic fibrosis. n=the number of isolates analyzed. Percentages indicate percentage of isolates with indicated errors and fractions in parentheses indicate numbers of isolates with the indicated errors. Type indicates the MIC range of the isolates. CAZ=ceftazidime. LVX=levofloxacin. MEM=meropenem. MIN=minocycline. TMP-SMX=trimethoprim-sulfamethoxazole. S=susceptible. I=intermediate. R=resistant. HD=Hardy Diagnostics. VME=very major error. ME=major error. MI=minor error. ND=not determinable for the error type. Red font indicates where acceptance criteria were not met.

**Table S4. Comparison of AD to BMD, CF and non-CF isolates.*^a^***

* a* Percentages indicate percentage of isolates with indicated errors and fractions in parentheses indicate numbers of isolates with the indicated errors. Type indicates the MIC range of the isolates. AD=agar dilution. BMD=broth microdilution. CF=cystic fibrosis. CAZ=ceftazidime. LVX=levofloxacin. MEM=meropenem. MIN=minocycline. TMP-SMX=trimethoprim-sulfamethoxazole. S=susceptible. I=intermediate. R=resistant. VME=very major error. ME=major error. MI=minor error. ND=not determinable for the error type. Red font indicates where acceptance criteria were not met.

**Table S5. Comparison of AD to BMD, non-CF isolates from respiratory and non-respiratory sources.*^a^***

* a* Percentages indicate percentage of isolates with indicated errors and fractions in parentheses indicate numbers of isolates with the indicated errors. Type indicates the MIC range of the isolates. AD=agar dilution. BMD=broth microdilution. CF=cystic fibrosis. CAZ=ceftazidime. LVX=levofloxacin. MEM=meropenem. MIN=minocycline. TMP-SMX=trimethoprim-sulfamethoxazole. S=susceptible. I=intermediate. R=resistant. VME=very major error. ME=major error. MI=minor error. ND=not determinable for the error type. Red font indicates where acceptance criteria were not met.

**Table S6. Comparison of ETEST to BMD, CF and non-CF isolates.*^a^***

**** *a* Percentages indicate percentage of isolates with indicated errors and fractions in parentheses indicate numbers of isolates with the indicated errors. Type indicates the MIC range of the isolates. BMD=broth microdilution. CF=cystic fibrosis. CAZ=ceftazidime. LVX=levofloxacin. MEM=meropenem. MIN=minocycline. TMP-SMX=trimethoprim-sulfamethoxazole. S=susceptible. I=intermediate. R=resistant. VME=very major error. ME=major error. MI=minor error. ND=not determinable for the error type. Red font indicates where acceptance criteria were not met.

**Table S7. Comparison of ETEST to BMD, non-CF isolates from respiratory and non-respiratory sources.*^a^***

* a* Percentages indicate percentage of isolates with indicated errors and fractions in parentheses indicate numbers of isolates with the indicated errors. Type indicates the MIC range of the isolates. BMD=broth microdilution. CF=cystic fibrosis. CAZ=ceftazidime. LVX=levofloxacin. MEM=meropenem. MIN=minocycline. TMP-SMX=trimethoprim-sulfamethoxazole. S=susceptible. I=intermediate. R=resistant. VME=very major error. ME=major error. MI=minor error. ND=not determinable for the error type. Red font indicates where acceptance criteria were not met.

**Supplemental Figures
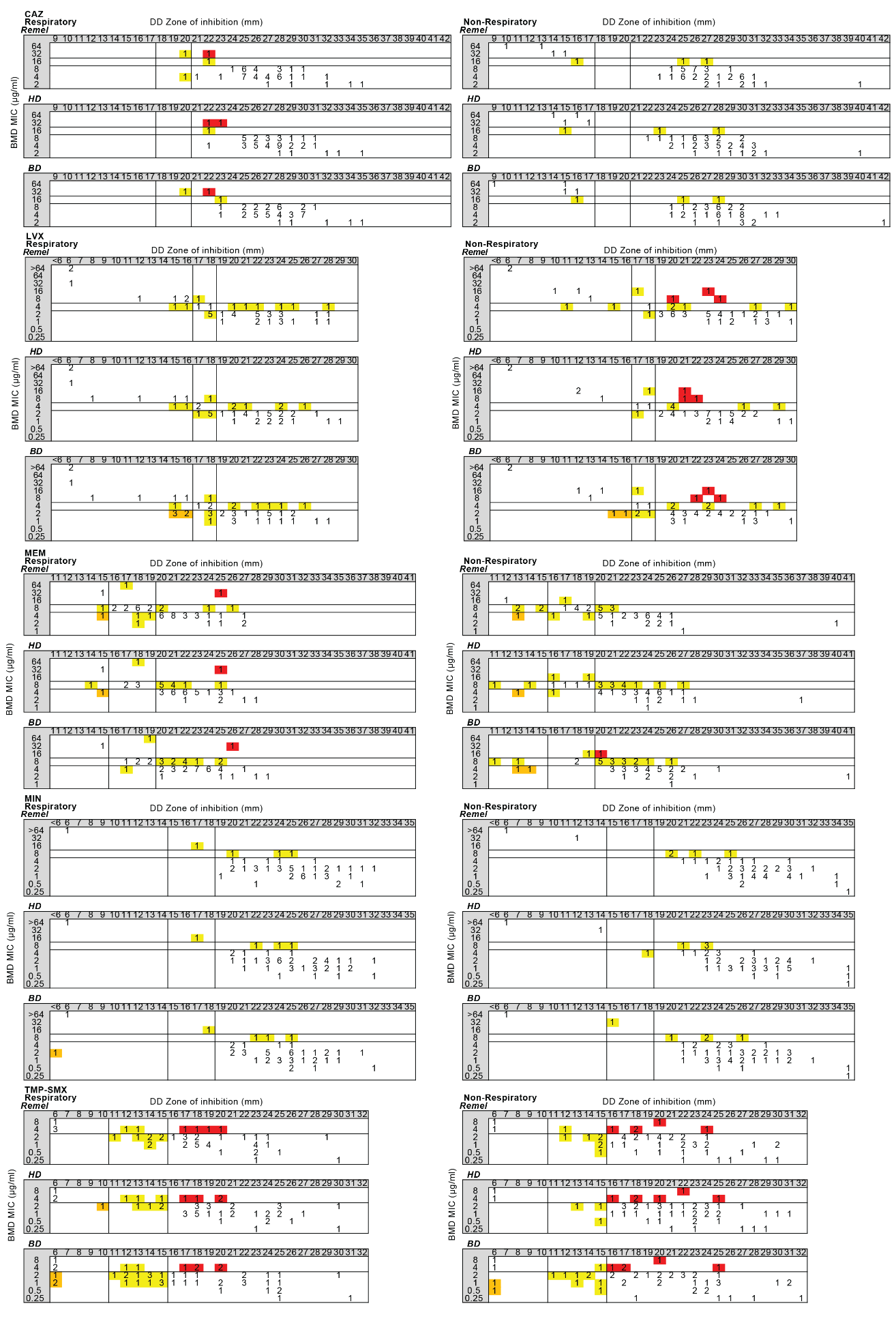
**

**Figure S1. Scattergrams comparing disk diffusion (DD) on 3 different Mueller Hinton Agar (MHA) brands to broth microdilution (BMD) for ceftazidime (CAZ), levofloxacin (LVX), meropenem (MEM), minocycline (MIN), and trimethoprim-sulfamethoxazole (TMP-SMX), non-cystic fibrosis (CF) isolates from respiratory (left) and non-respiratory (right) sources.** Scattergrams were generated by comparing the mode or median BMD MIC (y-axis) to DD zone diameter (x-axis) as measured on Remel, Hardy Diagnostics (HD), or BD MHA for non-CF isolates from respiratory and non-respiratory sources. Solid lines within each scattergram indicate the breakpoints applied. Red=very major error (VME), orange=major error (ME), and yellow=minor error (MI).

**
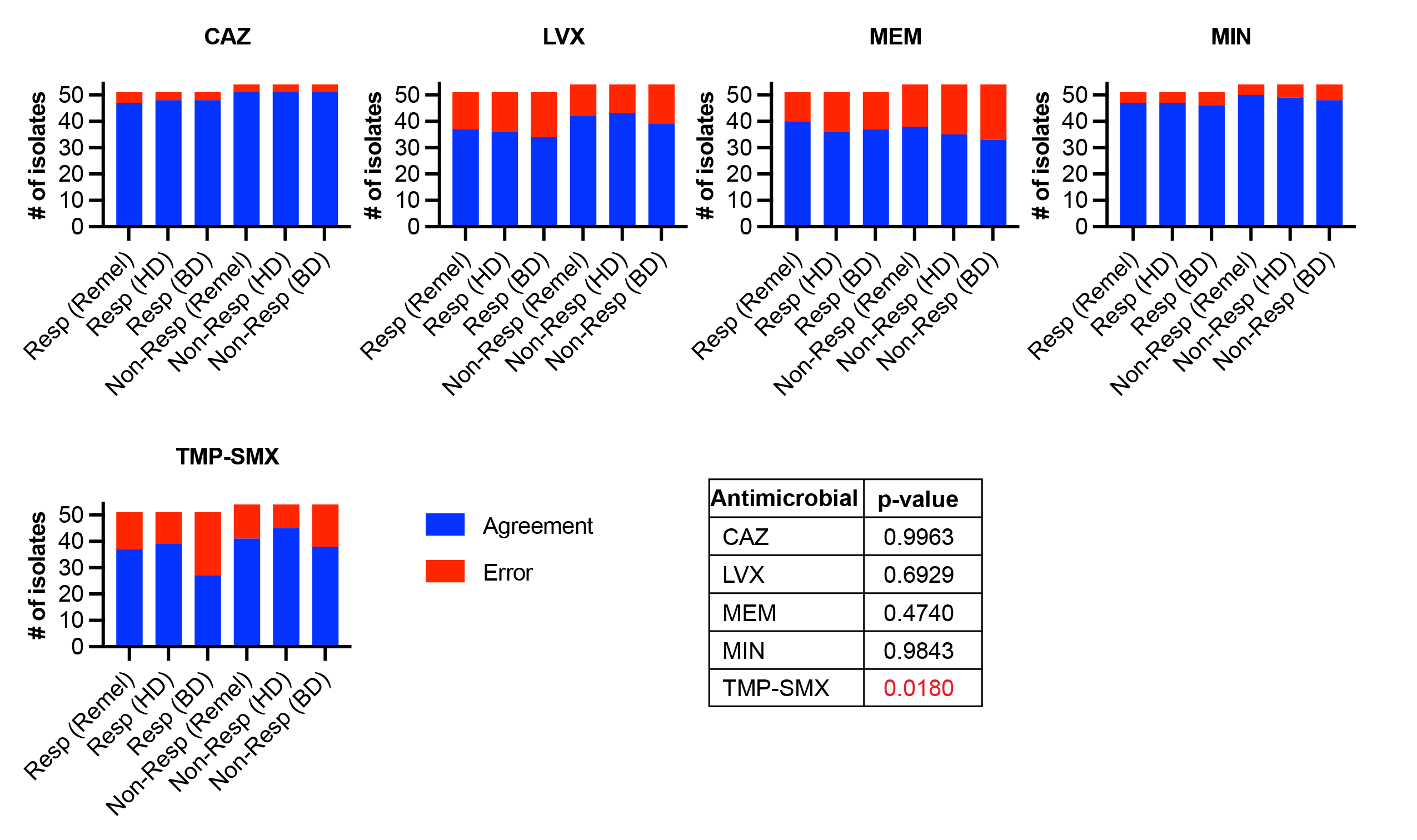
**

**Figure S2. Comparison of error rates for ceftazidime (CAZ), levofloxacin (LVX), meropenem (MEM), minocycline (MIN), and trimethoprim-sulfamethoxazole (TMP-SMX) disk diffusion (DD) as compared to broth microdilution (BMD) for non-cystic fibrosis (CF) isolates from respiratory and non-respiratory sources.** Very major errors (VMEs), major errors (MEs), and minor errors (Mis) for DD compared to BMD were summed for assays performed on 3 Mueller Hinton Agar (MHA) brands (Remel, Hardy Diagnostics (HD), and BD) and CF vs. non-CF isolates. The table summarizes Chi Square analyses for each drug, testing whether errors are under- or over-represented for DD performed on 3 different MHA brands for non-CF respiratory and non-respiratory isolates. p-value < 0.05 was considered significant. Significant values are shown in red font. Blue=agreement between methods; red=error between methods. #=number. Resp=respiratory. Non-resp=non-respiratory.

**
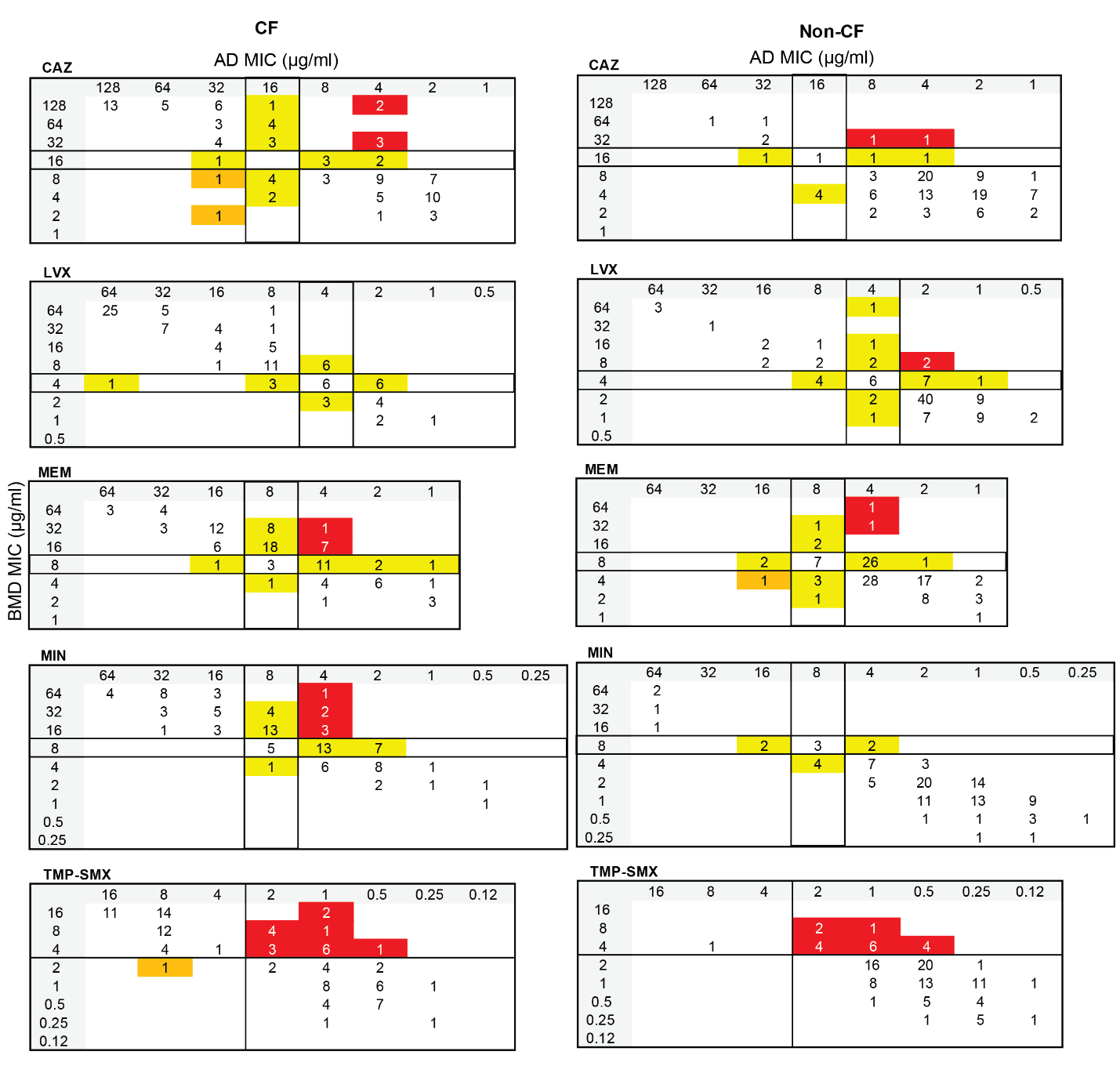
**

**Figure S3. Scattergrams comparing agar dilution (AD) to composite broth microdilution (BMD) for ceftazidime (CAZ), levofloxacin (LVX), meropenem (MEM), minocycline (MIN), and trimethoprim-sulfamethoxazole (TMP-SMX) for cystic fibrosis (CF) (left) and non-CF isolates (right).** Scattergrams were generated by comparing the composite BMD MIC (y-axis) to AD MIC (x-axis) for CF and non-CF isolates. Solid lines within each scattergram indicate the breakpoints applied. Red=very major error (VME), orange=major error (ME), and yellow=minor error (MI).

**
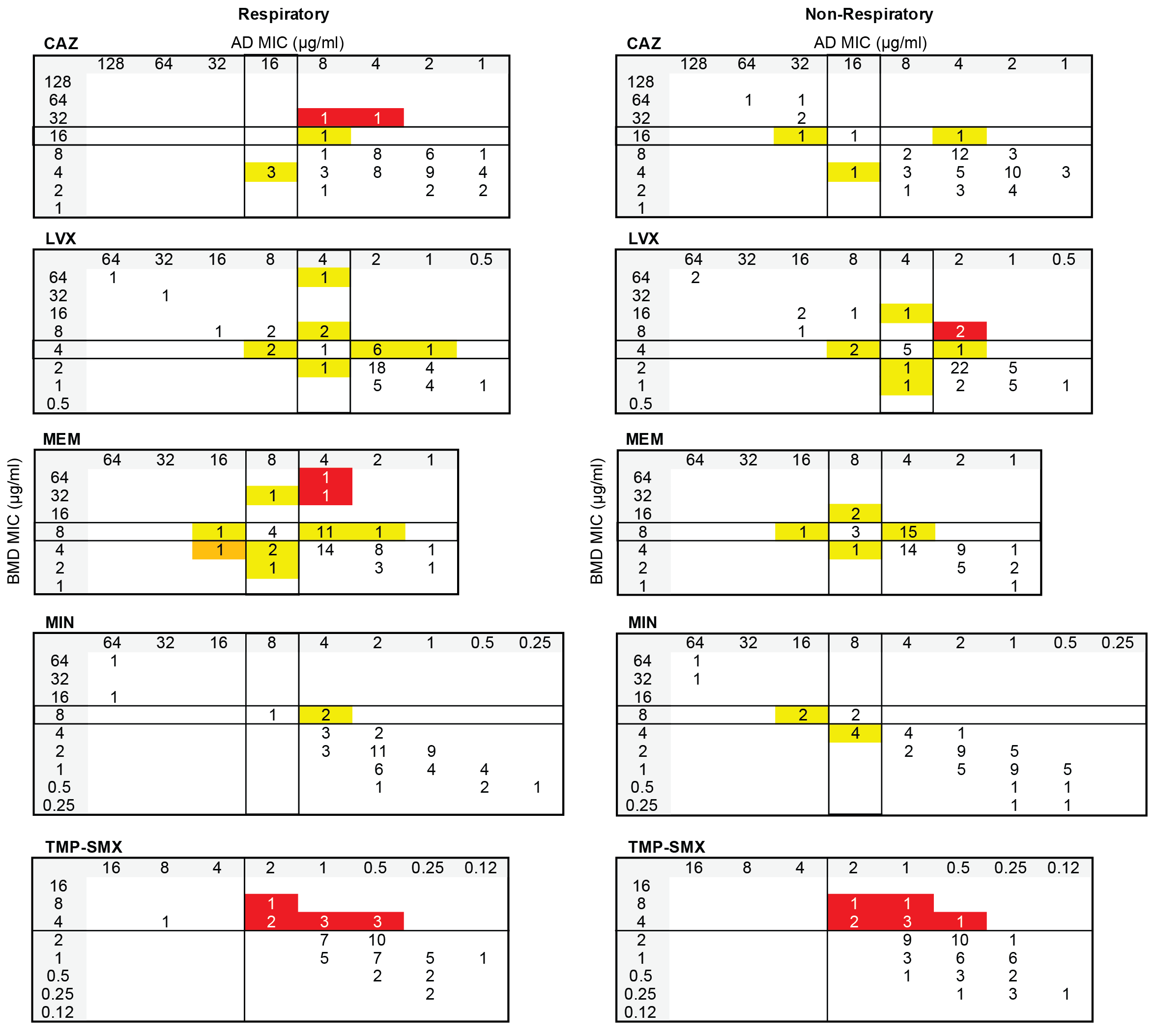
**

**Figure S4. Scattergrams comparing agar dilution (AD) to composite broth microdilution (BMD) for ceftazidime (CAZ), levofloxacin (LVX), meropenem (MEM), minocycline (MIN), and trimethoprim-sulfamethoxazole (TMP-SMX) for non-cystic fibrosis (CF) isolates from respiratory (left) and non-respiratory sources (right).** Scattergrams were generated by comparing the composite BMD MIC (y-axis) to AD MIC (x-axis) for non-CF isolates from respiratory and non-respiratory sources. Solid lines within each scattergram indicate the breakpoints applied. Red=very major error (VME), orange=major error (ME), and yellow=minor error (MI).


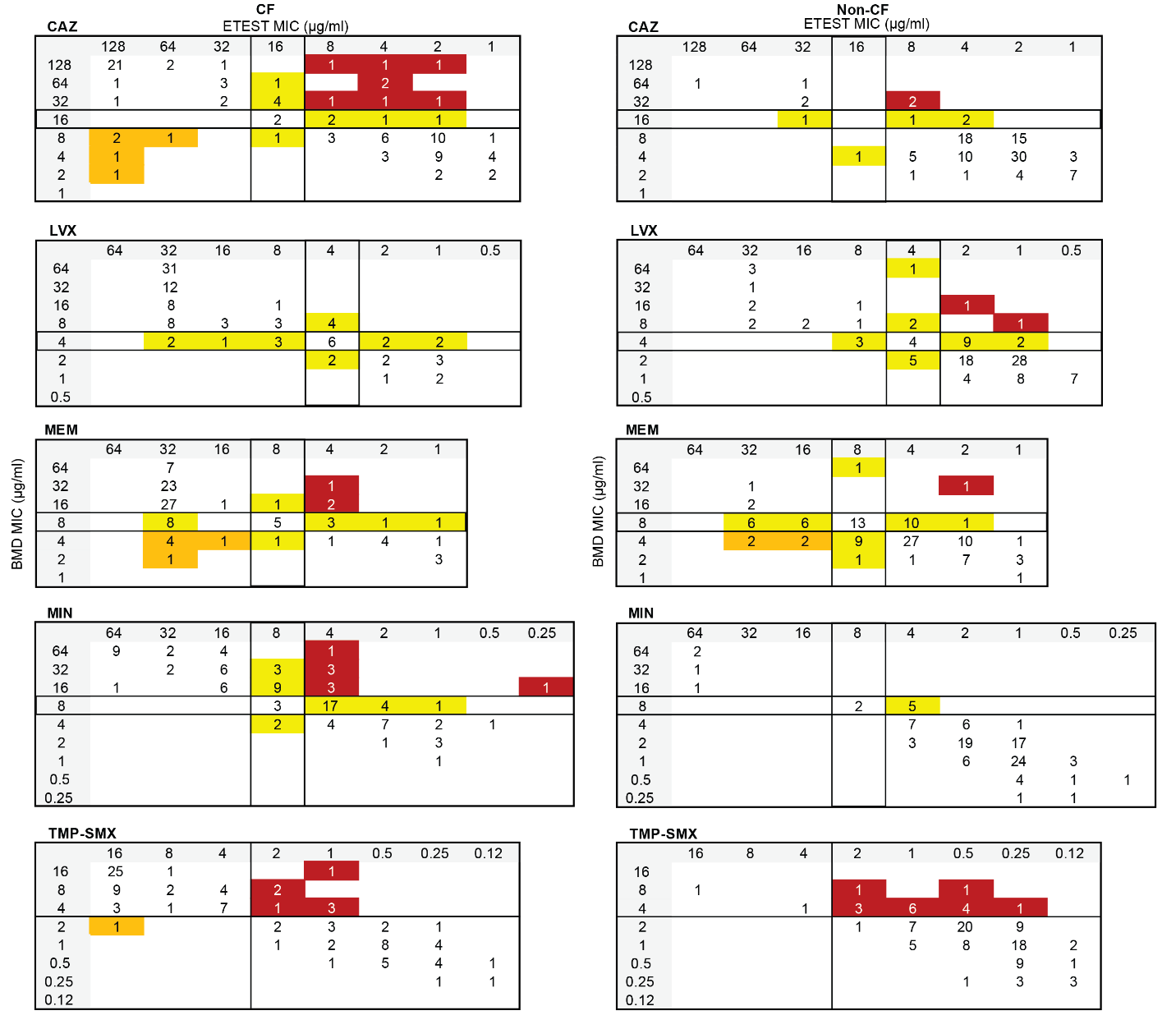


**Figure S5. Scattergrams comparing ETEST to composite broth microdilution (BMD) for ceftazidime (CAZ), levofloxacin (LVX), meropenem (MEM), minocycline (MIN), and trimethoprim-sulfamethoxazole (TMP-SMX) for cystic fibrosis (CF) (left) and non-CF isolates (right).** Scattergrams were generated by comparing the composite BMD MIC (y-axis) to ETEST MIC (x-axis) for CF and non-CF isolates. Solid lines within each scattergram indicate the breakpoints applied. Red=very major error (VME), orange=major error (ME), and yellow=minor error (MI).


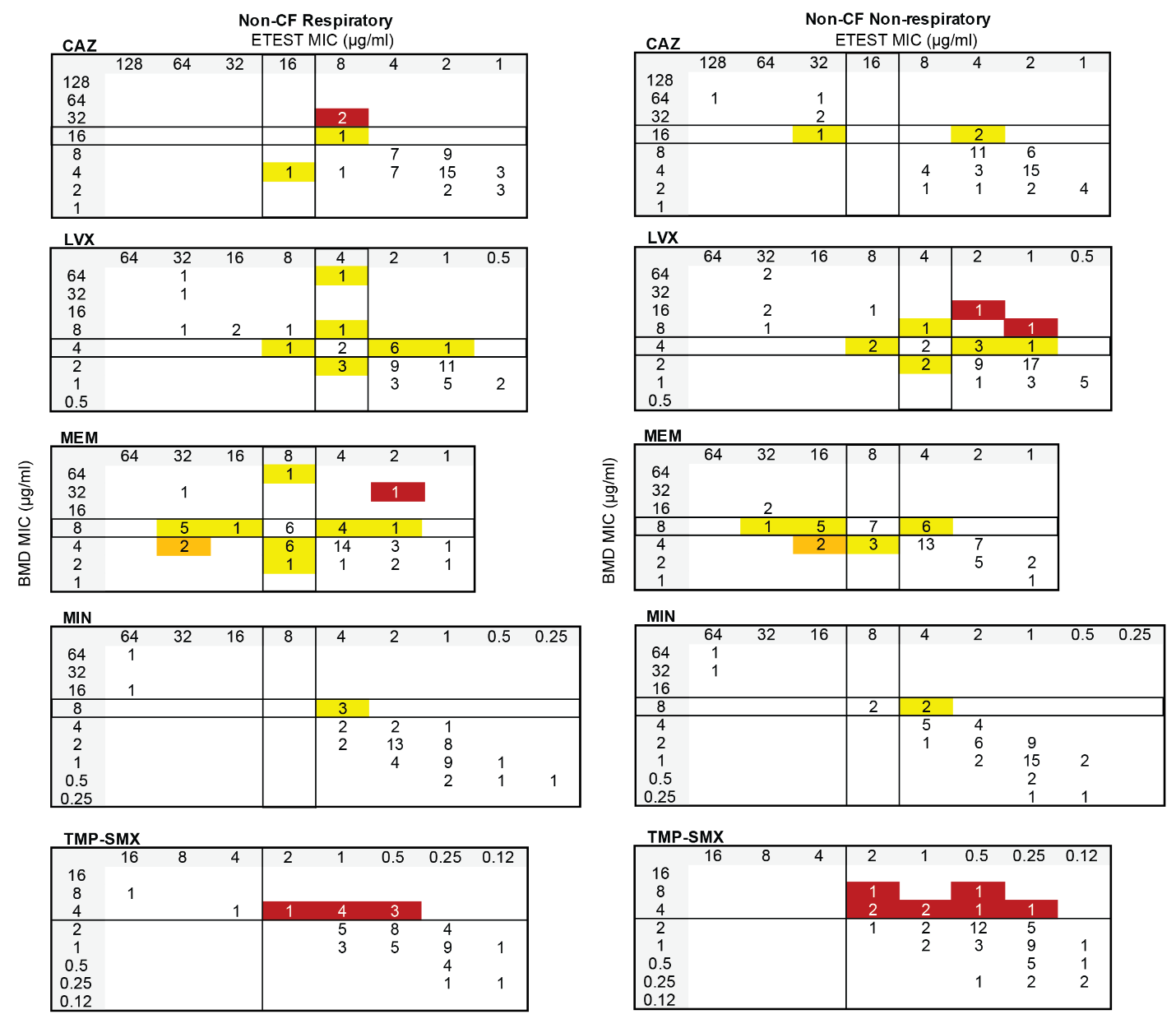


**Figure S6. Scattergrams comparing ETEST to composite broth microdilution (BMD) for ceftazidime (CAZ), levofloxacin (LVX), meropenem (MEM), minocycline (MIN), and trimethoprim-sulfamethoxazole (TMP-SMX) for non-CF isolates from respiratory (left) and non-respiratory sources (right).** Scattergrams were generated by comparing the composite BMD MIC (y-axis) to ETEST MIC (x-axis) for non-CF isolates from respiratory and non-respiratory sources. Solid lines within each scattergram indicate the breakpoints applied. Red=very major error (VME), orange=major error (ME), and yellow=minor error (MI).
